# A novel insertion mutation in atlastin 1 is associated with spastic quadriplegia, increased membrane tethering, and aberrant conformational switching

**DOI:** 10.1016/j.jbc.2021.101438

**Published:** 2021-11-19

**Authors:** Carolyn M. Kelly, Peter J. Zeiger, Vinodh Narayanan, Keri Ramsey, Holger Sondermann

**Affiliations:** 1 Department of Molecular Medicine, College of Veterinary Medicine, Cornell University, Ithaca, New York, USA; 2 Center for Rare Childhood Disorders, Translational Genomics Research Institute (TGen), Phoenix, Arizona, USA; 3 CSSB Centre for Structural Systems Biology, Deutsches Elektronen-Synchrotron DESY, Hamburg, Germany; 4 CSSB Centre for Structural Systems Biology, Christian-Albrechts-Universität zu Kiel, Kiel, Germany

**Keywords:** membrane tethering, neuropathy, GTPase, protein conformation, ACTH, adrenocorticotropic hormone, ATL1, atlastin-1, BSE, bundle signaling element, CP, cerebral palsy, DRP, dynamin-related protein, EEG, electroencephalogram, ER, endoplasmic reticulum, FRET, Förster resonance energy transfer, GED, GTPase effector domain, HSN, hereditary sensory neuropathy, HSP, hereditary spastic paraplegia, MLV, multilamellar vesicle, MRI, magnetic resonance imaging, TKO, triple-knockout, WT, wild-type

## Abstract

Hereditary spastic paraplegia (HSP) comprises a heterogeneous group of neuropathies affecting upper motor neurons and causing progressive gait disorder. Mutations in the gene *SPG3A/atlastin-1* (*ATL1*), encoding a dynamin superfamily member, which utilizes the energy from GTP hydrolysis for membrane tethering and fusion to promote the formation of a highly branched, smooth endoplasmic reticulum (ER), account for approximately 10% of all HSP cases. The continued discovery and characterization of novel disease mutations are crucial for our understanding of HSP pathogenesis and potential treatments. Here, we report a novel disease-causing, in-frame insertion in the *ATL1* gene, leading to inclusion of an additional asparagine residue at position 417 (N417ins). This mutation correlates with complex, early-onset spastic quadriplegia affecting all four extremities, generalized dystonia, and a thinning of the corpus callosum. We show using limited proteolysis and FRET-based studies that this novel insertion affects a region in the protein central to intramolecular interactions and GTPase-driven conformational change, and that this insertion mutation is associated with an aberrant prehydrolysis state. While GTPase activity remains unaffected by the insertion, membrane tethering is increased, indicative of a gain-of-function disease mechanism uncommon for ATL1-associated pathologies. In conclusion, our results identify a novel insertion mutation with altered membrane tethering activity that is associated with spastic quadriplegia, potentially uncovering a broad spectrum of molecular mechanisms that may affect neuronal function.

Hereditary spastic paraplegias (HSPs) are a heterogenous group of neurodegenerative disorders. They share disease presentation characterized by progressive spasticity and weakness of the legs caused by axonal degeneration of motor neurons, which begins at their distal ends (1, 2, 3, 4). Pathogenic mutations have been identified in at least 80 genes, denoted *SPG1-80*, due to the characteristic spastic gait and named in order of their identification (5, 6). The clinical manifestations of this disorder also vary significantly and are defined as either “pure” or “complex,” with pure cases primarily causing lower leg spasticity and complex cases involving additional neurological symptoms such as visual impairment, intellectual disability, epileptic seizures, and amyotrophy (2, 7). Incidence rates of HSPs are reported between 1.2 and 9.6 out of every 100,000 people (2, 4, 7, 8, 9), with inheritance modes ranging from autosomal dominant, autosomal recessive, X-linked recessive, and mitochondrial. Mutations in *SPG3A* (encoding human atlastin-1/ATL1) account for approximately 10% of all HSP cases, second only to *SPG4*, encoding spastin (10), and are the leading cause of early onset cases (11, 12). There have been 68 HSP-causing mutations identified in ATL1, with a majority being autosomal dominant and resulting in early onset and/or pure cases in patients (3, 5, 13).

The ATL1 protein belongs to the dynamin superfamily, members of which comprise a canonical, large GTPase (G) domain, and function in a variety of cellular pathways including vesicle fission, remodeling of organelle membranes, and immunity through antiviral activity (14). Other, structurally or functionally conserved domains within the dynamin superfamily include a stalk-like middle domain, a membrane-localization feature (*e.g.*, transmembrane domain, PH domain, lipid modification), and in many cases, a GTPase effector domain (GED)/bundle signaling element (BSE) for regulation of GTP hydrolysis. Canonically, coordinated GTPase activity in dynamin-related proteins (DRPs) is stimulated upon higher-order oligomerization including intra- and intermolecular interactions, orchestrated in part through the GED (15, 16, 17).

ATL1 is a resident ER enzyme, residing in the high-curvature tubules of the smooth ER, where it tethers and fuses tubules following GTPase-driven reaction cycles to generate and maintain the ER’s polygonal morphology (18, 19). The N-terminus of ATL1 is composed of the canonical large G domain followed by a flexible linker region and middle domain, facing the cytoplasm, followed by a short, membrane-associated wedge motif with preference for high-curvature ER tubules (20). At its C terminus, it contains a cytosolic, short, amphipathic helix that induces membrane disorder and is required for efficient fusion (21, 22). Unlike most DRPs, ATL1 catalyzes membrane fusion through hydrolysis-dependent homodimerization across *trans* membranes (23, 24, 25, 26). ATL1 is the minimal machinery necessary for membrane fusion (18) and formation of a reticular ER network *in vitro* (27). The N-terminal, cytoplasmic fragment comprising the G and middle domains acts as the catalytic core as it confers comparable GTPase activity as full-length ATL (28, 29) and functionally dimerizes with native ATL, evidenced by its action as a concentration- and GTPase-dependent inhibitor of membrane fusion *in vitro* (24, 29) and its dominant-negative effect on ER morphology when expressed in mammalian cells (29).

Most vertebrates encode three isoforms of ATL (ATL1-3), which retain high sequence homology (61–64%) and localize to the ER where they all catalyze membrane fusion (30). Despite these similarities, they vary in catalytic efficiency, ER spatial distribution, cellular fusion efficiency, and expression levels in tissues throughout the body, with ATL1 being primarily present in the brain (30, 31). ATL1 mutations associated with HSP have been shown to elicit variable GTPase activity and reduced membrane fusion capacity in cells, correlating with a reduction in three-way junctions visible in the ER network and other morphological changes of the ER (23, 24, 31, 32, 33, 34, 35). Many mutant alleles act as dominant-negative agents, causing phenotypes similar to those observed when all three ATL isoforms were deleted in mammalian cells (36). In addition to their role in HSP, familial mutations in ATL1 and ATL3 have been found to cause hereditary sensory neuropathy (HSN), another neurodegenerative disorder affecting sensory neurons (37, 38, 39).

Here we present a case of an early onset, complex HSP caused by a novel ATL1 mutation, where an asparagine residue is inserted into the middle domain between arginine residue 416 and tyrosine residue 417 (N417ins), the first reported clinical case caused by a whole codon insertion in ATL1. The proband presented with spastic quadriplegia and complex symptoms including generalized dystonia and thinning of the corpus callosum. Investigations into the structure and function of this novel ATL1 variant revealed that not only does the insertion mutant result in a stable protein, but it preserves many core functions, including GTP hydrolysis and nucleotide-dependent dimerization. Intriguingly, we discovered that this insertion mutant confers an uncommon gain-of-function phenotype *in vitro* and altered subcellular localization, which correlate with aberrant conformational switching of the enzyme and a complex HSP pathology.

## Results

### Patient case study

The proband is the first male child of a Hispanic and Caucasian/Asian couple born at term after an emergency caesarian section due to fetal heart rate decelerations. Birth weight was around 3600 g at 53 cm of height. Apgar scores were normal, and neither resuscitation nor intensive care support was required. Hospital discharge occurred at 3 days of age, and proband was specified normal as a neonate. Normal health ensued until 3 months, when adrenocorticotropic hormone (ACTH)-responsive infantile spasms developed. Magnetic resonance imaging (MRI) at the time was normal and an electroencephalogram (EEG) showed features of epilepsy. Cognitive function remained near normal, but in contrast, motor development was slow. Developmental hallmarks such as rolling over and crawling were reached at 6 and 14 months, respectively. At this point, motor development ceased, which resulted in the inability to walk. The patient was diagnosed with spastic quadriplegic cerebral palsy at 1.5 to 2 years. Cognitive development was near normal. He has been treated with intermittent injections of botulinum toxin, physical and occupational therapy, and has braces and splints.

**Figure 1:**
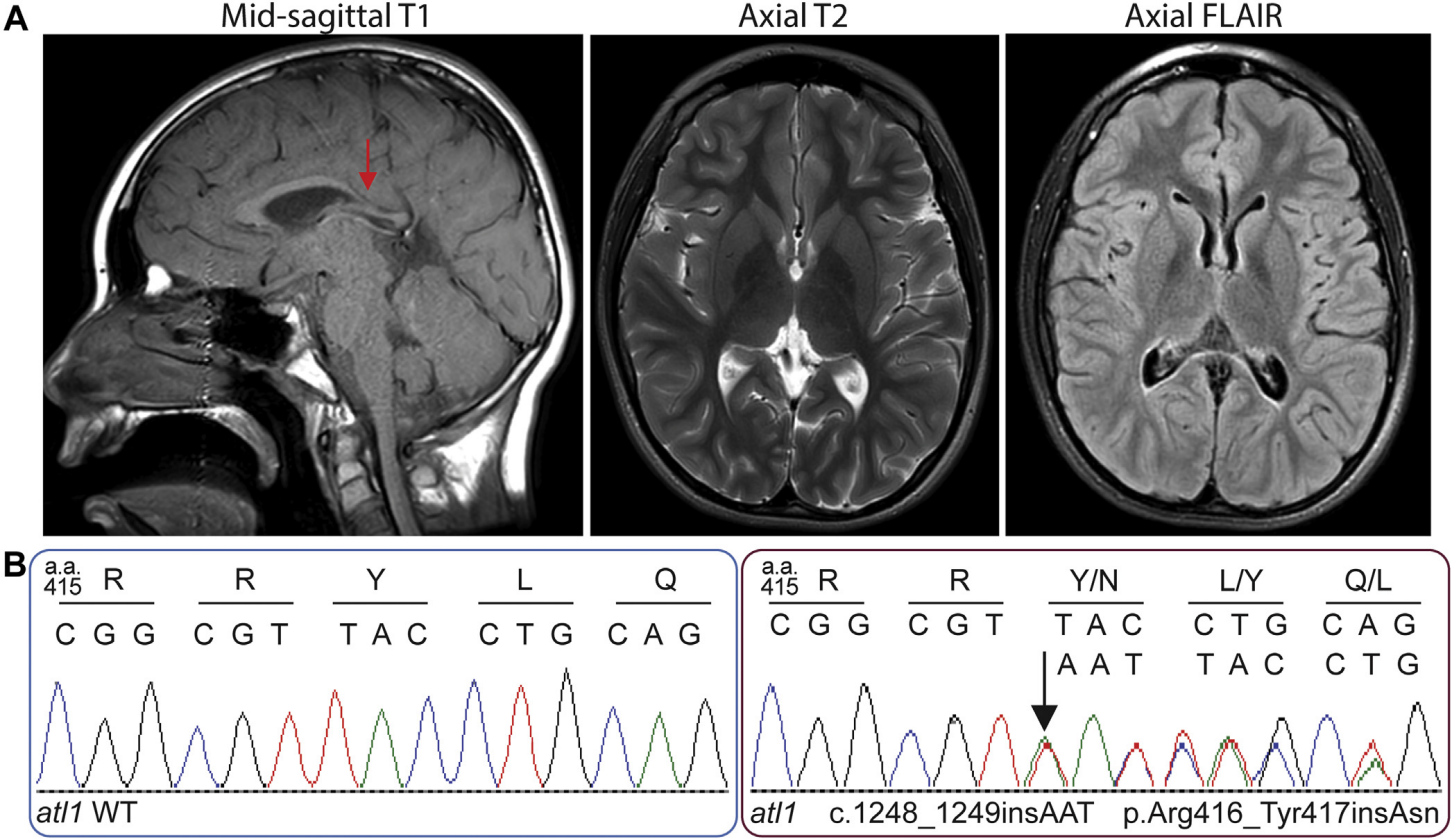
**MRI and Sanger sequencing of proband.***A*, MRI at 15 years showing thinning and down-sloping of the posterior body of the corpus callosum (*arrow* in *left panel*). *B*, Sanger sequencing chromatograms of *atl1* WT (*left*) and mutant (*right*) from the proband, starting at position 415 (amino acid)/1243 (nucleotide). The *arrow* indicates the location of the inserted codon (AAT) between position 1248 and 1249. Single letter amino acid and nucleotide codes indicated above.

At age 7, the proband had a feeding tube inserted and at age 15, a gastrostomy (G) tube but was able to eat smaller pieces of food and drink through a straw. Speech was slow but fairly clear. Limited movement existed in the arms and hands. Straightening of the arms at the elbows proved difficult, stiffness was present in the flexor group of the arm muscles, and a weak grip with contractures was present in both hands. Difficulties with movements and stiffness occurred in the extensor muscles of the legs. There was equinovarus posture of the feet and no movement. There was extensor tonus at the quadriceps, and mobility was enabled with the use of a motorized wheelchair. Cognitive development continued as exhibited through school attendance and normal intelligence. A follow-up brain MRI was reported as normal but showed thinning and down-sloping of the posterior body of the corpus callosum (Fig. 1*A*). This was unchanged in comparison to a prior imaging study done at age 3 years.

Using whole exome sequencing, a heterozygous *de novo* variant c.1248_1249insAAT:p.Arg416_Tyr417insAsn (referred to here as N417ins) was identified in exon 12 of the *ATL1* gene (NM_015915.4). This caused an in-frame insertion of a single asparagine residue in a nonrepeat region. *In silico* analysis supported a deleterious effect. The Combined Annotation Dependent Depletion score was 21.3. The variant was not found in gnomAD or Clinvar databases that catalog genomic variations, and in the latter case, link those to their relationship to human health (40, 41). Sanger sequencing of the variant was confirmed in the proband in a Clinical Laboratory Improvement Amendments (CLIA) laboratory (Fig. 1*B*).

### Mutation N417ins alters ATL1’s subcellular distribution

Previous studies have found that disease-causing mutations in ATLs can lead to ER morphologies ranging from large-scale aggregation to long, unbranched tubules or a relatively normal reticular structure depending on the mutation (31, 39, 42). Often, these mutations act in a dominant-negative fashion. Here we probed the impact of expressing the novel ATL1 N417ins mutation on the localization of ATL in both wild-type (WT) NIH-3T3 and NIH-3T3 ATL1/2/3 triple-knockout (TKO) mammalian cell lines (36). Usage of the TKO cells allowed us to investigate whether the mutation mimics WT ATL distribution in the absence of endogenous ATL isoforms, while use of the WT cells would reveal whether endogenous ATL rescues potential variations of localization of the mutant.

**Figure 2:**
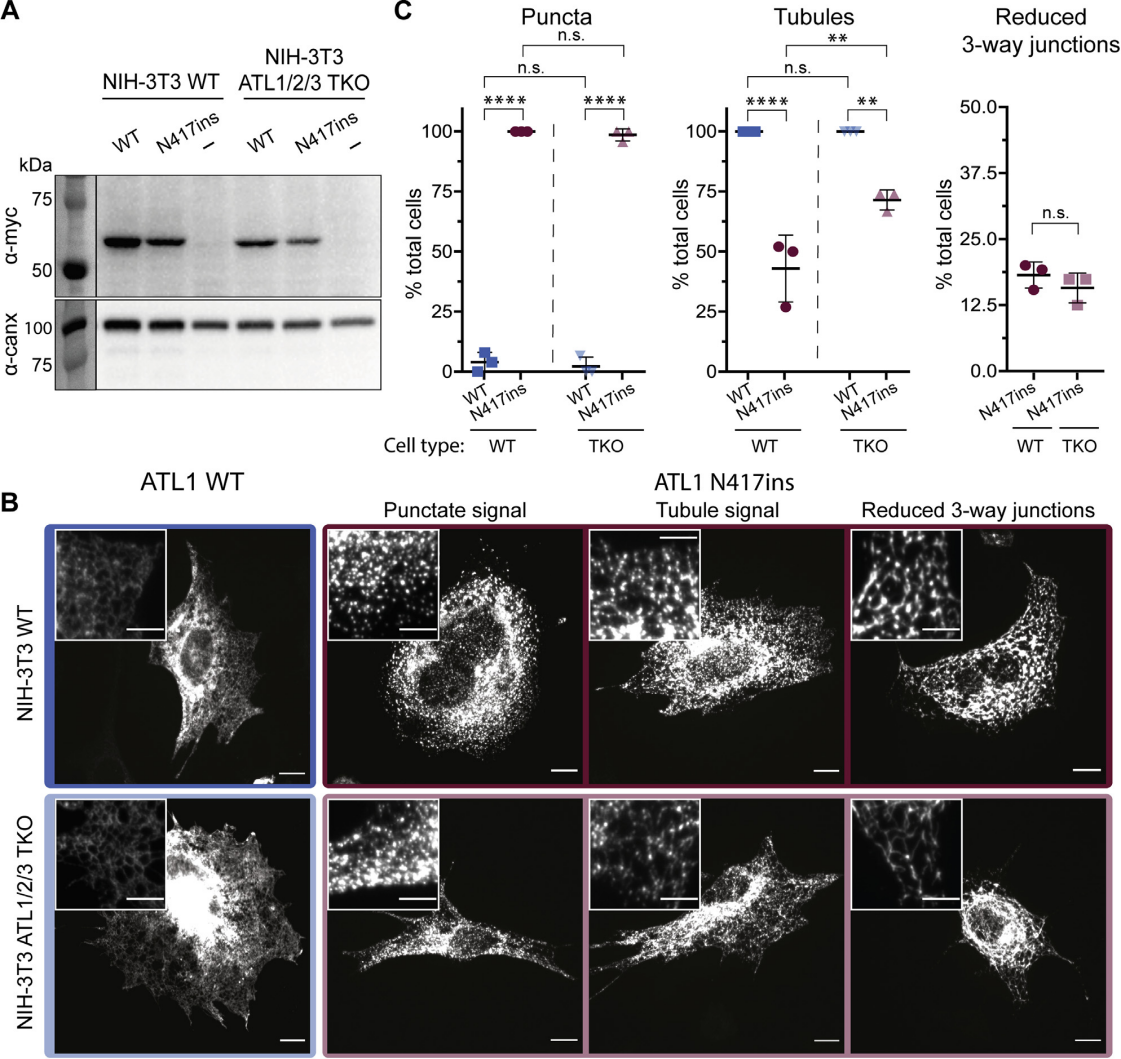
**Effect of N417ins on ATL1’s subcellular localization.***A*, ATL1 expression levels in transiently transfected NIH-3T3 (WT) and NIH 3T3 ATL1/2/3 triple knockout (TKO) cells (36). Recombinantly expressed, myc-tagged ATL1 WT and N417ins were detected by Western blotting with a c-myc-specific primary antibody; empty pcDNA vector was included as a control. Total protein concentration from lysates was normalized before loading, and the signal from an anti-calnexin Western blot was used as a loading control. *B*, immunofluorescence of NIH-3T3 WT (*top*) and NIH-3T3 ATL1/2/3 TKO (*bottom*) transiently transfected with either ATL1 WT^myc^ (*blue*/*light blue box*) or N417ins^myc^ (*maroon*/*pink box*) and probed with α-c-myc antibodies for imaging with confocal microscopy. Representative images of reported phenotypes in cells expressing ATL1 N417ins are labeled along the *top* (Punctate, tubule, or reduced three-way junctions). Scale bar = 10 μm; inset scale bar = 5 μm. *C*, quantification of observed ATL1^myc^ localization phenotypes including punctate (*left*), tubular (*middle*), and reduced visible three-way junctions (*right*); categories are not mutually exclusive. A minimum of 70 cells were imaged and quantified from three individual experiments (except NIH-3T3 ATL1/2/3 TKO expressing ATL1 wild-type, which had 46 cells). Three biological replicates were carried out for each condition. *Symbols* represent percent of total cells in each replicate exhibiting that phenotype. *Middle bar* shows the mean of the replicates and error bars show the SD. One-way ANOVA tests established statistical significance.

We first compared protein abundance of exogenously expressed ATL1 WT and N417ins mutant variant with a C-terminal myc-tag. Immunoblotting verifies that both constructs express in WT and TKO cells (Fig. 2*A*). To assess impact of the mutant on ATL distribution, we carried out immunofluorescence staining in NIH-3T3 WT and TKO cells exogenously expressing ATL1 WT and N417ins and imaged the cells with confocal microscopy. Wild-type protein localization matches results reported previously, with ATL1 evenly decorating a highly reticular, tubular network (Figs. 2*B* and S1) (31). Strikingly, the N417ins variant primarily localizes to many puncta and apparent clusters throughout the cell (Figs. 2*B* and S1). This punctate localization pattern is observed both in WT and TKO cell lines, indicating it is an effect independent of endogenous ATL expression. Although the N417ins-containing protein appears to have altered ER localization, the ER remains largely intact and the N417ins mutant colocalizes with an ER marker as determined by cotransfection of U2OS cells with ^mCherry^SEC61B (Fig. S1).

To establish phenotypic trends in WT and TKO cell lines expressing either WT or mutant ATL, we categorized distribution patterns of each cell to provide a semiquantitative assessment as follows. Of the NIH-3T3 cells quantified, 100% and 98.6% of N417ins transfected cells had puncta while only 4% and 2.2% of WT-transfected showed this feature, in WT and TKO cells, respectively (Fig. 2*C*). The primary variation among N417ins transfected cells was the extent of discernable tubule signal, which ranged from (1) no tubules (individual puncta that do not appear to trace to a reticular pattern), (2) puncta that clearly trace along tubule patterns, (3) puncta with faint homogenous tubule signal, to (4) strong tubule signal (with or without puncta) (Fig. 2*B*). We determined the percentage of observed cells with any discernable tubule signal (categories 2–4) and found that 100% of exogenous ATL1 WT generated a tubular signal to only 43% and 71.5% of N417ins-transfected cells, in WT and TKO cells, respectively (Fig. 2*C*). It is noteworthy that while the percentage of cells with punctate N417ins variant signal in WT and TKO cells was not significantly different, the difference of cells with tubular localization of the exogenous protein was statistically significant. Lastly, of the cells with any level of tubule signal, 18.2% and 15.8% of N417ins transfections had visibly reduced three-way junctions, in WT and TKO cells, respectively (Fig. 2*C*). This phenotype has been observed with expression of some HSP variants and catalytically inactive ATL1 mutants (31, 32, 34, 36, 42). Overall, distribution of the ATL1 N417ins mutant is not dependent of the presence of endogenous ATL WT isoforms, correlating with the heterozygosity of the mutation in the proband.

### GTP hydrolysis and oligomerization by ATL1 are not affected by the N417ins mutation

**Figure 3:**
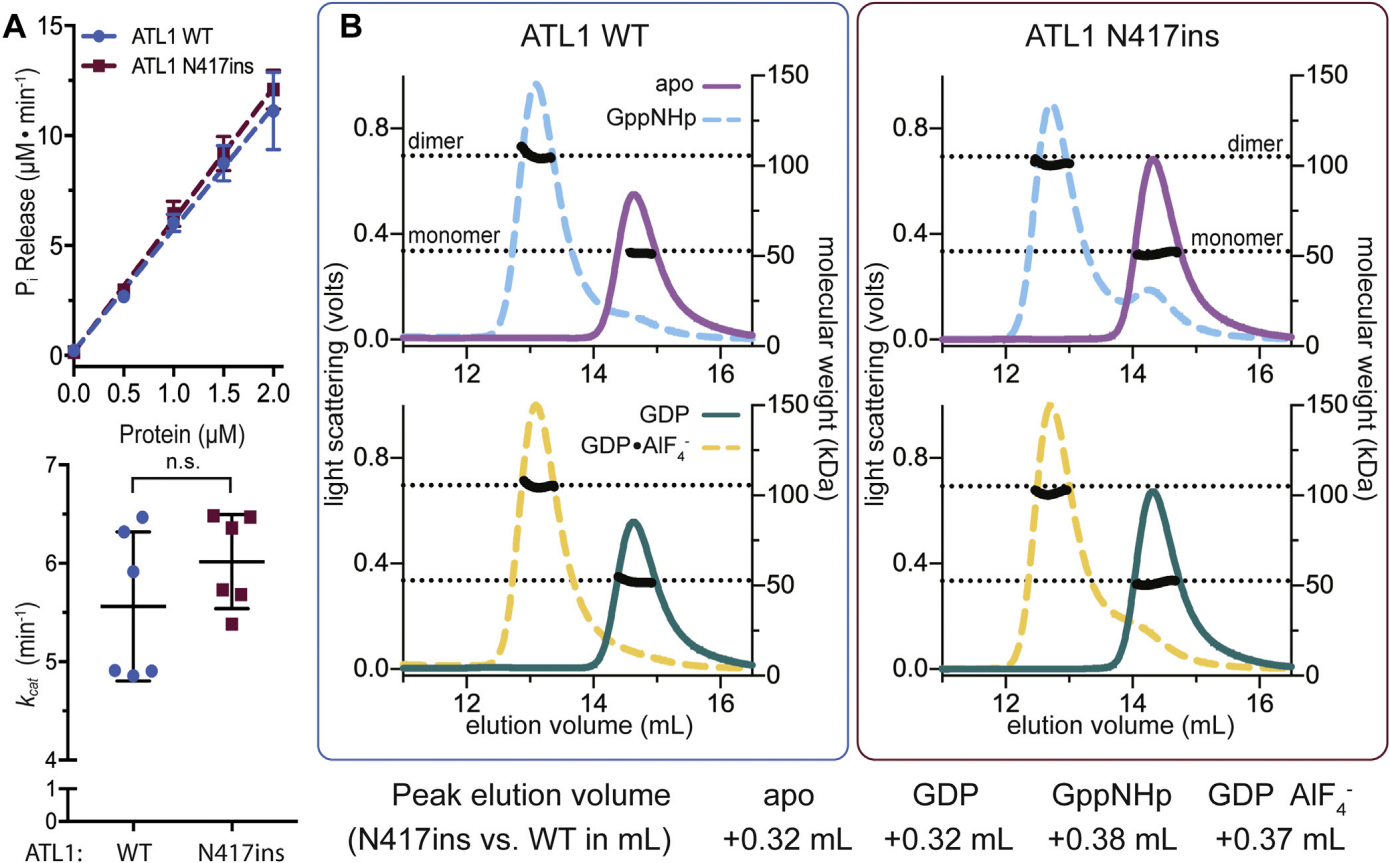
**GTP hydrolysis and nucleotide-dependent dimerization of ATL1 N417ins.***A*, GTP hydrolysis measured *via* release kinetics of P_i_ for ATL1 WT and N417ins catalytic cores across protein concentrations (0–2 μM) are shown (*top panel*). *Symbols* represent the mean of two biological and three technical replicates with error bars showing standard deviations (SD). Apparent turnover rates (*k*_*cat*_) were calculated from the P_i_-release kinetics for each replicate with the *middle bar* representing the mean and error bars showing SD. An unpaired *t* test evaluated significance (*p* = 0.0858). *B*, nucleotide-dependent oligomerization. Chromatograms from SEC-MALS experiments with either ATL1 WT (*left*) or N417ins (*right*), with all *colored lines* representing light scattering signal in volts denoted on the *left axis* (GppNHp, apo, GDP•AlF4^−^, and GDP), and *black lines* indicating calculated molecular weight in kDa (*right axis*). *Dotted lines* represent the theoretical molecular weights of the monomer and dimer (52.9 kDa and 105.8 kDa for N417ins; 52.8 kDa and 105.6 kDa for WT). For each nucleotide condition, the volume at the maximum light scattering signal for WT was subtracted from that of N417ins and the shift is indicated in ml (*bottom text*).

Having established a pronounced cellular phenotype, we set out to characterize the effect of the ATL1 N417ins mutation on the protein’s molecular mechanism. It has been established that the N-terminal soluble module of ATL1 consisting of the conserved G and middle domains, which we refer to as the catalytic core, is sufficient to carry out GTP hydrolysis and nucleotide-dependent oligomerization (23, 28, 29). The fragment also interacts with the full-length protein in a GTPase-dependent manner, suggesting that its functional cycle tracks that of the native protein (24, 29, 43). For those reasons, we can employ the soluble, near-full-length catalytic core as a proxy for the native protein in biochemical studies reporting on intrinsic properties of ATL. Both WT and mutant proteins expressed to comparable levels in *E. coli*, and purifications produced similar overall protein yields, suggesting no major impact of the sequence alteration on protein stability. As a first test, we used an established spectrophotometric assay to measure the release of inorganic phosphate (P_i_) over time during GTP hydrolysis for both WT and mutant protein. The catalytic activity was the same for both proteins with no statistically significant difference in *k*_*cat*_ values of ATL1 WT and ATL1 N417ins (apparent rates of 5.6 ± 0.18 min^−1^ for WT and 6.0 ± 0.13 min^−1^ for the N417ins mutant protein) (Fig. 3*A*).

A key characteristic of ATLs is their ability to form dimers upon GTP hydrolysis, a state that can be trapped by incubating the proteins with nonhydrolyzable GTP analogs (GppNHp or GTPγS) or a transition state analog (GDP•AlF_4_^−^) (23, 24, 44). In contrast, the ATL catalytic core remains monomeric in the absence of nucleotides or in the presence of GDP (23, 44). This pattern of dimerization is crucial in conferring ATL’s ability to tether and fuse membranes and is retained across all isoforms and orthologs, even extending to yeast Sey1p and mitofusins (28, 29, 44, 45, 46, 47, 48). We tested whether the novel disease mutation affects this conserved pattern by using size exclusion chromatography in tandem with multiangle light scattering (SEC-MALS) (49). As established previously, this assay reports on the molecular weight and conformation of proteins in solution. We found that, like ATL1 WT, the N417ins mutant protein remained a monomer in apo and GDP conditions and dimerized in the presence of GppNHp and GDP•AlF_4_^−^ (Fig. 3*B*). Only a minor fraction of the mutant protein eluted as a monomer upon incubation with GppNHp, and less so with GDP•AlF_4_^−^, suggesting that the mutation could affect dimer stability to some minor extent. Another notable deviation from WT was the retention time on the column, with N417ins eluting 0.32 ml earlier as a monomer and 0.37 to 0.38 ml earlier as a dimer (Fig. 3*B*). Since we saw no change in calculated molecular weight accompanying these shifts in retention time, they indicate deviations in the hydrodynamic radius of the proteins and their oligomers, likely due to conformational differences.

### Structure of ATL1 with the N417ins mutation bound to GDP•AlF_4_^−^

**Figure 4:**
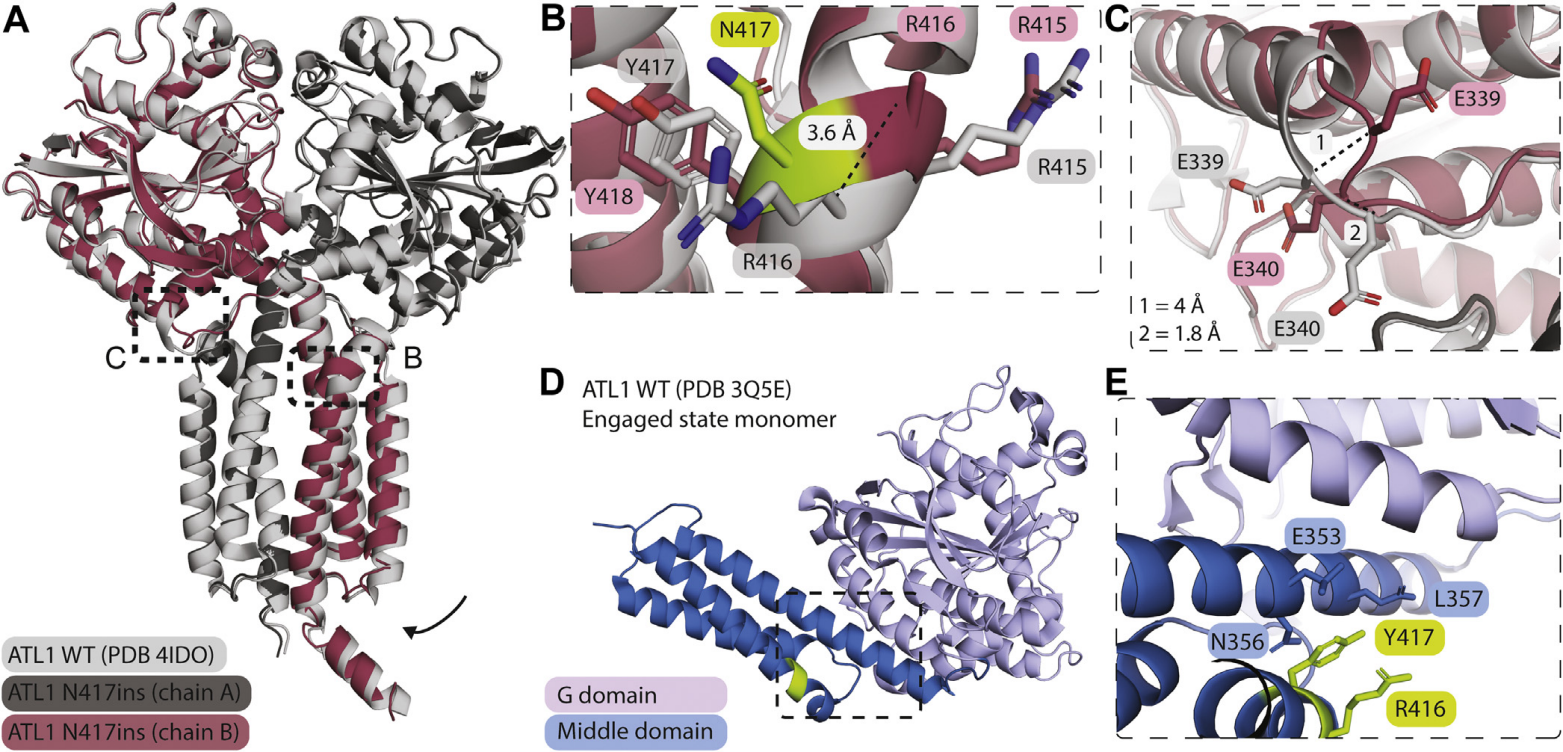
**Structure of the ATL1 N417ins crossover dimer bound to GDP•AlF**_**4**_^**−**^**.***A*, structural overview. ATL1 N417ins (*dark gray* and *maroon*) superimposed with ATL1 WT (*light gray*; PDB 4IDO) each bound to GDP•AlF4^−^ and Mg^2+^. *Black boxes* indicate boundaries of zoom-in views in *B* and *C*. *Arrow* indicates slight rotation of mutant middle domain. *B*, zoom-in view of the insertion mutation in chain B. Each residue is labeled (number of N417ins residues were adjusted after residue N417 to accommodate the extra residue). The distance spanning the corresponding R416 Cα positions between the WT and N417ins structures is indicated (3.6 Å). N417 is colored in *green* while other residues were colored as in *A*. *C*, magnified view of the transition between the G domain and linker region of chain B, with both E339 and E340 of each structure labeled. Distances between the same residue in both structures is indicated in the *lower left corner*. *D*, structure of the monomeric, engaged ATL1 WT state (PDB 3Q5E) bound to GDP and Mg^2+^. The residues directly pre- and proceeding the N417ins mutation (R416 and Y417) are indicated in *green*. The *black box* indicates the region shown in *E*. *E*, position of the insertion mutation. R416 and Y417 lie directly adjacent to middle and G domain helices integral in engaged state formation.

Having not seen a significant difference in the intrinsic activity of ATL1 with the N417ins mutation, we embarked on solving the crystal structure in order to determine the extent of conformational disruptions in the protein by the mutation. The protein with the insertion mutation crystallized in the presence of the transition state analog GDP•AlF_4_^−^ and Mg^2+^, diffracting to a resolution of 1.9 Å. The structure was solved by molecular replacement in space group P2_1_2_1_2_1_ using isolated G and middle domain fragments as the search models, identifying two molecules in the asymmetric unit (Table 1). The novel, dimeric structure aligns with that of a corresponding ATL1 WT protein bound to GDP•AlF_4_^−^ (25) with an rmsd value of 0.386 Å, both structures depicting a tight crossover dimer of ATL1’s catalytic core (Fig. 4*A*). The individual protomers superimpose nearly exactly with the corresponding chains in the WT structure (rmsd chain A = 0.294 Å; rmsd chain B = 0.514 Å).

**Table 1:** Data collection and refinement statistics^a^

Data collection and refinement parameters	ATL1 N417ins
Wavelength	0.9686
Resolution range	46–1.9 (1.97–1.90)
Space group	P 21 21 21
Unit cell	49.1 115.0 184.0 90 90 90
Total reflections	604,079 (52,441)
Unique reflections	82,941 (8042)
Multiplicity	7.3 (6.5)
Completeness (%)	99.75 (97.83)
Mean I/sigma(I)	8.79 (1.00)
Wilson B-factor	21.59
R-merge	0.2319 (1.691)
R-meas	0.2496 (1.835)
R-pim	0.09145 (0.6986)
CC1/2	0.992 (0.335)
CC^a^	0.998 (0.708)
Reflections used in refinement	82,909 (8019)
Reflections used for R-free	2000 (194)
R-work	0.1734 (0.3049)
R-free	0.2201 (0.3070)
CC (work)	0.966 (0.673)
CC (free)	0.953 (0.754)
Number of non-hydrogen atoms	7860
macromolecules	6901
ligands	12
solvent	947
Protein residues	847
RMS (bonds)	0.007
RMS (angles)	0.86
Ramachandran favored (%)	98.70
Ramachandran allowed (%)	1.30
Ramachandran outliers (%)	0.00
Rotamer outliers (%)	1.33
Clashscore	3.42
Average B-factor	24.66
macromolecules	23.81
ligands	14.39
solvent	31.52
Number of TLS groups	6

a Statistics for the highest-resolution shell are shown in parentheses.

The inserted N417 residue is surface exposed within the middle domain’s third helix. In this state, the ⍺-helical rung containing the insertion accommodates the additional residue by bulging out slightly, resulting in minimal long-range structural changes. According to the DSSP secondary structure assignment, the inserted residue results in formation of π-helix from residues 415 to 419 (50, 51). The primary variation between WT and insertion-mutant structures lies within the middle domain dimer, where a slight translation is observed toward the second protomer’s middle domain within the dimeric assembly of the mutant, with the middle domain interface tightening by ∼0.7 to 1.2 Å (interface-proximal) to 1.0 to 1.6 Å (solvent-proximal). The only exception to this observed domain translation is residue R416, directly preceding the insertion mutation, which shifts 3.6 Å in its C⍺ position and rotates outward (Fig. 4*B*).

The only other notable structural change occurs at the interface of the G domain and linker region of chain B at residues E339 and E340. Here we observe a 180° flip of the E339 side chain and a 4 Å shift of the corresponding Cα′s and ∼60° side chain rotation and 1.8 Å shift in the E340 Cα. This disturbance remains localized to these two residues (and specific to chain B) but appears to have a subtle effect on where the last helix of the G domain terminates (Fig. 4*C*).

Although we only obtained the structure of ATL1 N417ins in a crossover dimer state so far, the GDP-bound, wild-type monomer structure we determined previously for ATL1 places the insertion region adjacent to the first helix of middle domain (Fig. 4, *D* and *E*). This region is of functional importance since it is proximal to the G-middle domain interface in this engaged state, characteristic for the GDP-bound conformation and likely involved in the allosteric coupling between the two domains (25, 44).

### ATL1 N417ins has markedly increased membrane tethering rates *in vitro*

**Figure 5:**
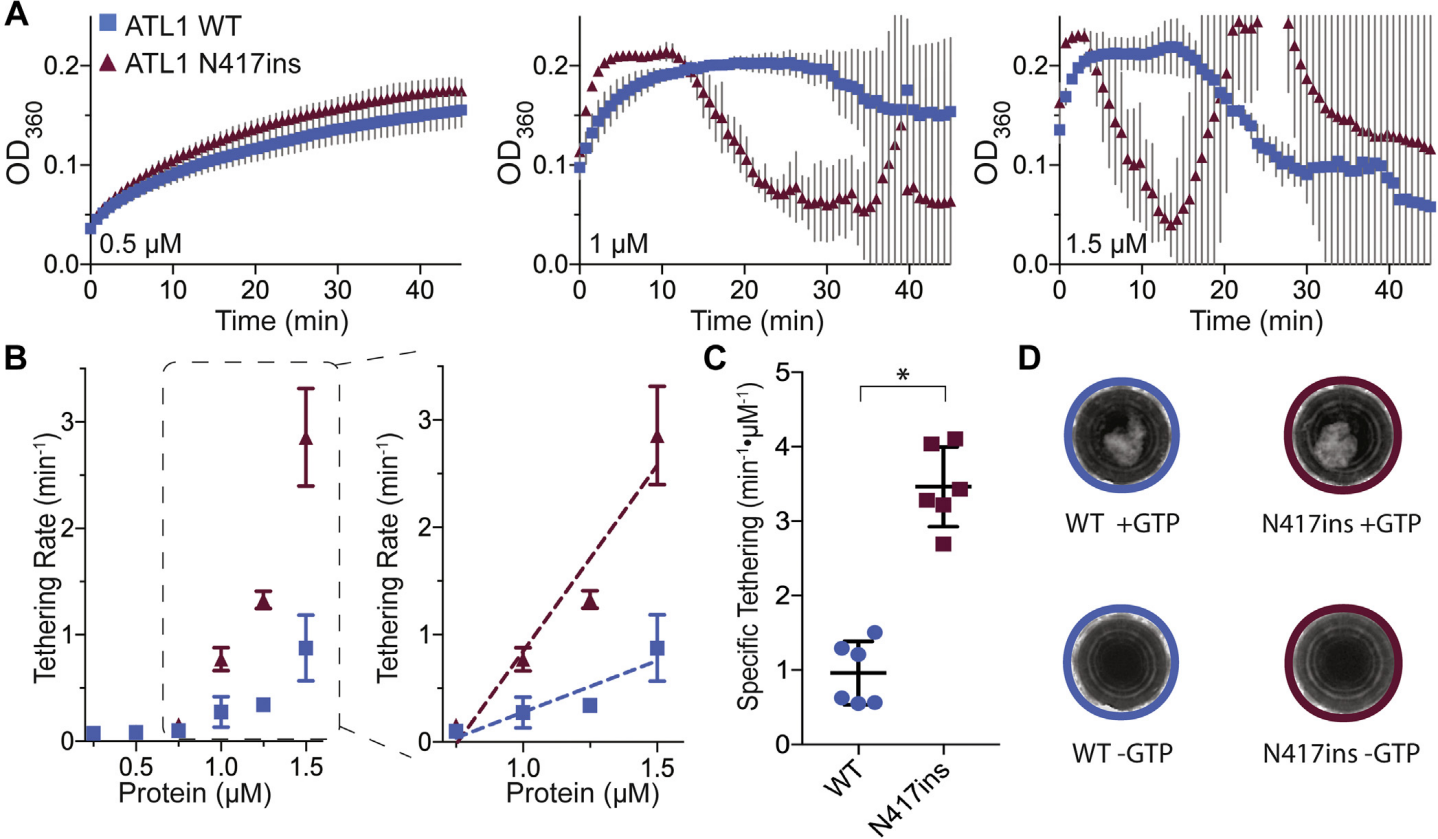
**The N417ins mutation increases membrane tethering efficiency of the ATL1 catalytic-core fragment.***A*, select tethering reactions at increasing protein concentrations (between 0.5 and 1.5 μM protein) for ATL1 WT (*blue*) and N417ins (*maroon*). Each point is the average of two biological and three technical replicates, with *gray* error bars for the SD. *B*, initial tethering rates (min^−1^) were calculated for each ATL1 concentration, displayed as the mean and SD across replicates. The *inset* (*right panel*) shows concentrations used to calculate values plotted in *C* (reactions with 0.75–1.5 μM protein). *C*, specific tethering rates (min^−1^ μM^−1^) for each construct’s technical and biological replicates. An unpaired *t* test was used to determine the significance between the WT and mutant rates (*p* = 0.0237). *D*, images (top views of the individual wells of a 96-well plate; well diameter = 5 mm) of tethering reactions after 45 min ± GTP at 1.5 μM.

Since we saw no effect of the mutation on GTP hydrolysis or nucleotide-dependent dimerization and the crossover state structure is largely unaffected, we next investigated what effect the mutant had on membrane-related functions of ATL1. Since full-length, human ATL1 has not been reconstituted in a membrane fusion-competent form, we tested for this function by using an accepted vesicle-tethering assay utilizing the soluble catalytic core of ATL1 (26). Tethering events are en route to fusion and may signify a native ATL function (26, 52). To assay for GTPase-dependent membrane tethering, purified WT or mutant ATL1 catalytic cores with a decahistidine tag at their C-termini were loaded onto vesicles containing 1% (molar ratio) Ni(NTA)-modified lipids. The resulting presentation of the soluble ATL1 domains mimics the topology of the full-length, transmembrane protein. Flotation assays confirmed that both WT and mutant protein partitioned to the vesicles fraction (Fig. S2). Upon the addition of GTP, continual vesicle tethering caused an increase in solution turbidity, which was measured spectrophotometrically (at OD_360_). Figure 5*A* shows representative tethering reactions of ATL1 WT and N417ins at increasing concentrations from 0.5 to 1.5 μM (additional reactions were carried out between 0.25 μM and 1.5 μM). At all concentrations, the signal increases over time as expected. At higher concentrations (1 μM and above), the signal drops after reaching a plateau at the maximum (OD_360_ ∼ 0.2) followed by erratic fluctuations. This pattern is the product of large, macroscopically visible vesicle clumps (Fig. 5, *A* and *D*). The effect was reversed upon addition of EDTA or imidazole (Fig. S2). Additional control reactions were carried out with GDP or without MgCl_2_, which did not support vesicle tethering (Fig. S2).

To compare tethering of WT and mutant protein, the initial tethering rates of each reaction were calculated across protein concentration (Fig. 5*B*). From this, it is apparent that there are two linear regions of increasing tethering rates (0.25 μM–0.75 μM and 0.75 μM–1.5 μM), with ATL1 N417ins demonstrating both higher initial tethering rates and a faster rate of increase per protein concentration compared with WT protein (ATL1 at 0.959 ± 0.28 and N417ins at 3.46 ± 0.65 min^−1^ μM^−1^) (Fig. 5*C*).

### The GDP-bound catalytic core of ATL1 N417ins is more susceptible to limited proteolysis than wild-type protein

**Figure 6:**
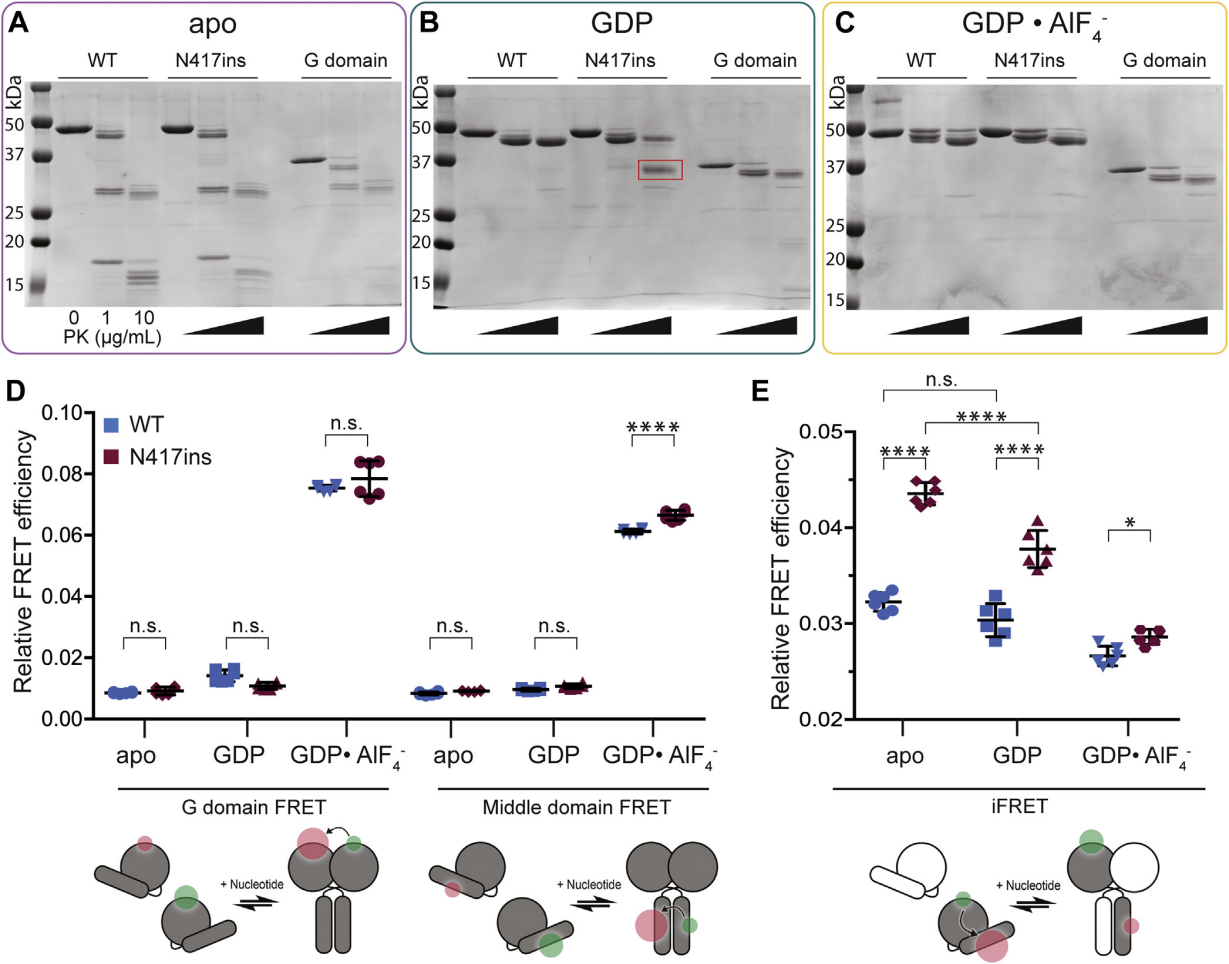
**Altered conformation of the monomeric ATL1 N417ins.***A*–*C*, SDS-PAGE gels show limited proteolysis reactions with increasing concentrations of proteinase K (PK) from 0 to 10 μg/ml and 2 μM ATL1 (catalytic core of ATL1 WT and N417ins; purified G domain) for 15 min on ice. Reactions were incubated either in the absence of nucleotide (*A*), with 2 mM GDP (*B*), or with GDP•AlF_4_^−^ (*C*). The *red box* in (*B*) indicates a band of interest for the mutant and molecular weight markers are indicated on the *left* of each gel. *D*, intermolecular FRET between G domains (*left*) and middle domains (*right*) for ATL1 WT (*blue*) and N417ins (*maroon*) in the presence of indicated nucleotides (X-axis). Relative FRET efficiencies shown by *symbols* for three technical and two biological replicates for each condition (n = 6) with mean shown by the *middle bar* and SD by error bars. *E*, iFRET within ATL1 WT (*blue*) or N417ins (*maroon*) carried out with a 1:10 excess of unlabeled protein. Conditions and statistics are the same as in (*D*). *Cartoons* at the *bottom* of (*D* and *E*) represent experimental setups.

With the unusual pattern of *in vitro* activity observed for the N417ins variant with only subtle deviations in the crossover dimer structure, we sought to determine if conformational changes in the predimer state could account for increased tethering rates and observed cellular phenotypes. To test this, we used proteinase K (PK) at low concentrations (0, 1, and 10 μg/ml) in a limited proteolysis experiment. We expected that if variations in conformation exist, it could result in different banding patterns on an SDS-PAGE gel based on relative accessibility of cleavage sites. For each reaction, the catalytic core of ATL1 WT and ATL1 N417ins, as well as the ATL1 G domain (residues 1–339), was preincubated with the indicated nucleotide prior to addition of PK. In the apo state (Fig. 6*A*), both WT and mutant proteins have a single primary band in the absence of PK and at 10 μg/ml have a series of bands clustered near 30 kDa and a number of bands under ∼20 kDa, which corresponds with size and relative amounts of bands seen in the G domain control.

However, when bound to GDP, the WT protein is largely protected from degradation, with the most prominent band only a few kDa below the intact protein (Fig. 6*B*). While the N417ins mutant does contain a band of the same size at the highest PK concentration, the most pronounced band appears as a diffuse band at ∼35 kDa (Fig. 6*B*, red box). This band corresponds very closely to the size of the G domain alone (residues 1–339 assemble to a protein of 39 kDa). Since ATL1 undergoes substantial conformational changes pertaining to the relative positions between the G and middle domains (23), the G domain is a rational candidate for this band. When samples are preincubated with the transition state analog GDP•AlF_4_^−^, both WT and N417ins are largely protected from degradation (Fig. 6*C*), indicating that the relevant cleavage sites for the given protease concentrations are protected in the tight crossover-dimer state of both proteins (Fig. 4; (25)). These reactions were also carried out with the protein loaded on vesicles to determine if these cleavage patterns would be altered in the presence of a membrane, but we found banding patterns nearly identical to the reactions lacking liposomes (Fig. S3).

### Inter- and intramolecular FRET indicate conformational variations of the monomeric ATL1 N417ins

The results of the structural analysis and limited proteolysis suggested changes in conformation between ATL1 WT and the N417ins variant, which could give insight to the disease mechanism. To further describe these, we used a Förster resonance energy transfer (FRET) assay to assess relative distances within the homodimer and the relative intramolecular distance between the G and middle domain within a single protomer, an approach we established previously for ATLs (25, 44). Briefly, intermolecular FRET was carried out with either the WT or mutant catalytic core with all surface-exposed cysteine residues mutated to alanine and a cysteine introduced either on the surface of the G or middle domain. For intramolecular FRET, two cysteine residues were introduced strategically in the middle and G domain of a single protein. These cysteine residues were labeled with a maleimide-conjugated AlexaFluor dye with peak excitation at either 488 nm (donor) or 647 nm (acceptor). For intermolecular G or middle domain FRET, protein was labeled with either donor or acceptor AlexaFluor dye in separate reactions; for intramolecular FRET experiments, both cysteine residues were labeled stochastically in one reaction with both dyes present.

Relative FRET efficiencies were measured for steady-state intermolecular interactions between the G domains or the middle domains after preincubation with various nucleotides (Fig. 6*D*). All reactions were equilibrated for 1 h before measurement based on previously established nucleotide equilibration times (25, 44). Based on the measured intermolecular FRET signals (Fig. 6*D*), there was no significant difference between ATL1 WT and N417ins with the exception of the middle domain of the N417ins mutant bound to GDP•AlF_4_^−^, which had a small but significant increase for the mutant indicating a shorter average Förster distance. This is in agreement with the middle domain translation observed in our structure (Fig. 4*A*).

A previously described intramolecular FRET (iFRET) sensor was used next to measure changes in distances between the G and middle domains within a monomer in the same nucleotide-bound states (44). In this system, we expect the engaged monomer to have a higher relative signal, which would decrease as the G domain is released and a crossover dimer is formed. A 1:10 ratio of labeled to unlabeled protein was used to minimize contribution of intermolecular signal across a homodimer. As predicted, we see that for ATL1 WT the apo state has the highest iFRET with a slight decrease upon GDP binding and a larger decrease upon GDP•AlF_4_^−^ binding (Fig. 6*E*). However, we see several notable differences for the protein with the N417ins mutation. In both the apo and GDP-bound states, there was a significant increase in signal compared with that observed with the WT protein under identical conditions (*p* < 0.0001 for each). In addition, we see lower relative iFRET signals for GDP•AlF_4_^−^ as is seen with the WT. While the decrease in FRET signal between apo and GDP for WT was not significant (loss of 0.002 in relative FRET efficiency, *p* = 0.056), there was a much greater loss for the N417ins mutant (loss of 0.006, *p* < 0.0001), indicating a notable conformational change upon nucleotide binding that was not observed with ATL1 WT.

## Discussion

Here we have identified a novel mutation in ATL1 that causes a complex form of spastic paraplegia involving all four extremities of the proband. We demonstrate that the mutant protein has an increased membrane tethering potential *in vitro* despite unaltered GTPase activity, correlating with cellular defects in the subcellular distribution of the full-length protein. To date, there have been at least 68 HSP disease-causing mutations identified in the *SPG3A/ATL1* gene, with a majority being missense mutations, a small number caused by single nucleotide insertions or deletions, and only one di-nucleotide insertion (53, 54). The disease-causing mutation described in this study is the first report of a whole-codon insertion in *ATL1*.

The proband in this study has generalized weakness and bilateral spasticity of his legs. The symptoms appear to be more severe with generalized dystonia and difficulty moving both the arms and legs. The involvement of arm and grip weakness, difficulty straightening out at his elbows, and limited hand movements indicate that the pathology is more than spastic paraplegia. Tendon reflexes were brisk at the biceps and present at the knees, which suggested that the patient does not have a generalized motor or sensory neuropathy. We propose that the N417ins variant contributes to the child’s symptoms and describes a new phenotype that could be a part of a spectrum of disorders resulting from mutations in the *ATL1* gene.

When ATL1 HSP mutants are expressed in mammalian cells, a variety of morphological aberrations affecting the ER have been observed, including aggregated bundles and globules, pervasive puncta, long, unbranched tubules, and a shift in sheet-to-tubule ratios (31, 32, 34, 35, 42, 55). Expression of the novel ATL1 N417ins variant in NIH-3T3 cells both with and without endogenous ATL present resulted most prominently in the localization of the protein to puncta and small clusters, likely along ER tubules and at tubule junctions considering the colocalization of ATL1 N417ins with an ER marker in U2OS cells. Of the observed phenotypes, the only notable difference between the WT and TKO cells was the increased percentage of cells with visible tubules in the cells lacking all endogenous ATL isoforms (Fig. 2*C*). This observation suggests that the ATL1 protein with the insertion mutation may retain membrane tethering and/or fusion activity, but the effect of the mutation on the spatial distribution of the protein and ER structure may be dominant in the presence of endogenous ATL isoforms.

In order to probe what may cause these cellular disruptions and disease pathogenesis, we assessed the effect of the N417ins mutation on the activity and structure of the ATL1 protein. Previous reports that took a similar approach in characterizing HSP mutations have found varying levels of deviations in GTP hydrolysis rates, extent of nucleotide-dependent dimerization, and structural conformations as assessed by X-ray crystallography (23, 24, 32, 33, 35, 56). We found that the innate GTP hydrolysis rate of the ATL1 N417ins mutant variant does not differ significantly from its WT counterpart (Fig. 3*A*). Similarly, nucleotide-dependent oligomerization of the protein appeared unaffected by the mutation (Fig. 3*B*). However, differences in hydrodynamic radius as read out by the peak elution volume in size-exclusion chromatography of the mutant protein compared with WT suggest an impact of the mutation on the global conformation of the protein. This result mirrored altered inter- and intramolecular distances observed in the ATL1 protein carrying the mutation by equilibrium FRET measurements (Fig. 6, *D* and *E*). While the crystal structure of a soluble transition-state dimer of ATL1 only showed subtle deviations compared with a corresponding WT structure (25), a protein stability assay revealed a particular protease sensitivity of the mutant protein in its GDP-bound state (Fig. 6, *A*–*C*). One feature observed in the crystal structure of the protein with the insertion mutation is the transition from an α-to π-helix spanning residues 415 to 419, which includes the insertion. A previous link has been drawn between evolutionary α-to π-helix transitions caused by a single residue insertions and gain-of protein function (57), in the case of ATL1 N417ins being implicated in human pathogenesis.

The structural alterations correlate with an impact of the mutation on ATL1’s function. Specifically, *in vitro* vesicle tethering using the catalytic core as a proxy for the full-length protein’s function on the ER membrane showed a significantly increased vesicle tethering rate of the mutant protein compared with the corresponding WT (Fig. 5). This apparent gain-of-function effect of the mutation is in contrast to reports on other HSP-associated ATL1 variants that usually act as loss-of-function alleles, at least for those where a deviation from WT activity was detected (23, 24, 32, 33, 35, 56). Although speculative at this point, the higher tethering rate *in vitro* agrees with a model in which the corresponding full-length protein supports membrane fusion function in cells while also showing a more concentrated localization to puncta that may present sites of hyper-tethering/fusion (58). Microscopic investigations at higher spatial and temporal resolution will be required to corroborate the potential link between tethering rates supported by the soluble core fragment and the function of the full-length proteins in cells. Our observations also raise subsequent questions about the mechanism leading to the altered cellular distribution of the N417 insertion mutant and potential impacts on ER structure or dynamics while the protein’s innate GTPase activity is unaltered by the mutation.

Although the proteolytic sensitivity of the mutant protein in the GDP-bound state implicates the prefusion state of ATL1 in the pathogenic mechanism, the crystal structure of the transition state also shows subtle conformational changes that were corroborated by FRET-based experiments. The residues directly pre- and proceeding the insertion lie adjacent to the region of the middle domain α-helix 1 that interacts with the G domain. It has been previously established that the G domain of the prehydrolysis monomer interacts with α-helix 1 of the middle domain (23, 24), and its release on a biologically relevant time scale is dependent upon GTP binding and hydrolysis (44). The undocking of the middle domain from the G domain is a prerequisite for protein dimerization, membrane tethering, and fusion (25, 29, 44). In this context, we hypothesize that the N417ins mutation may alter the prehydrolysis state in such a way that the propensity of the middle domain being released from the G domain increases, which could lead to faster membrane tethering without altering the innate activity of the enzyme. Such a model is consistent with our observation that the insertion mutation leads to increased cleavage between G and middle domains in the catalytic-core fragment upon limited proteinase K treatment. Whether the middle domain is destabilized by the mutation and more prone to further degradation compared with the WT middle domain is currently an open question. In summary, the limited proteolysis and FRET studies indicate a conformational disruption of the apo and GDP-bound monomer and potential destabilization of the engaged state with the middle domain docked at the G domain. Membrane tethering rates are increased likely due to this dysregulation of release of the G and middle domains upon GTP hydrolysis and initiation of *trans* dimer formation.

**Figure 7:**
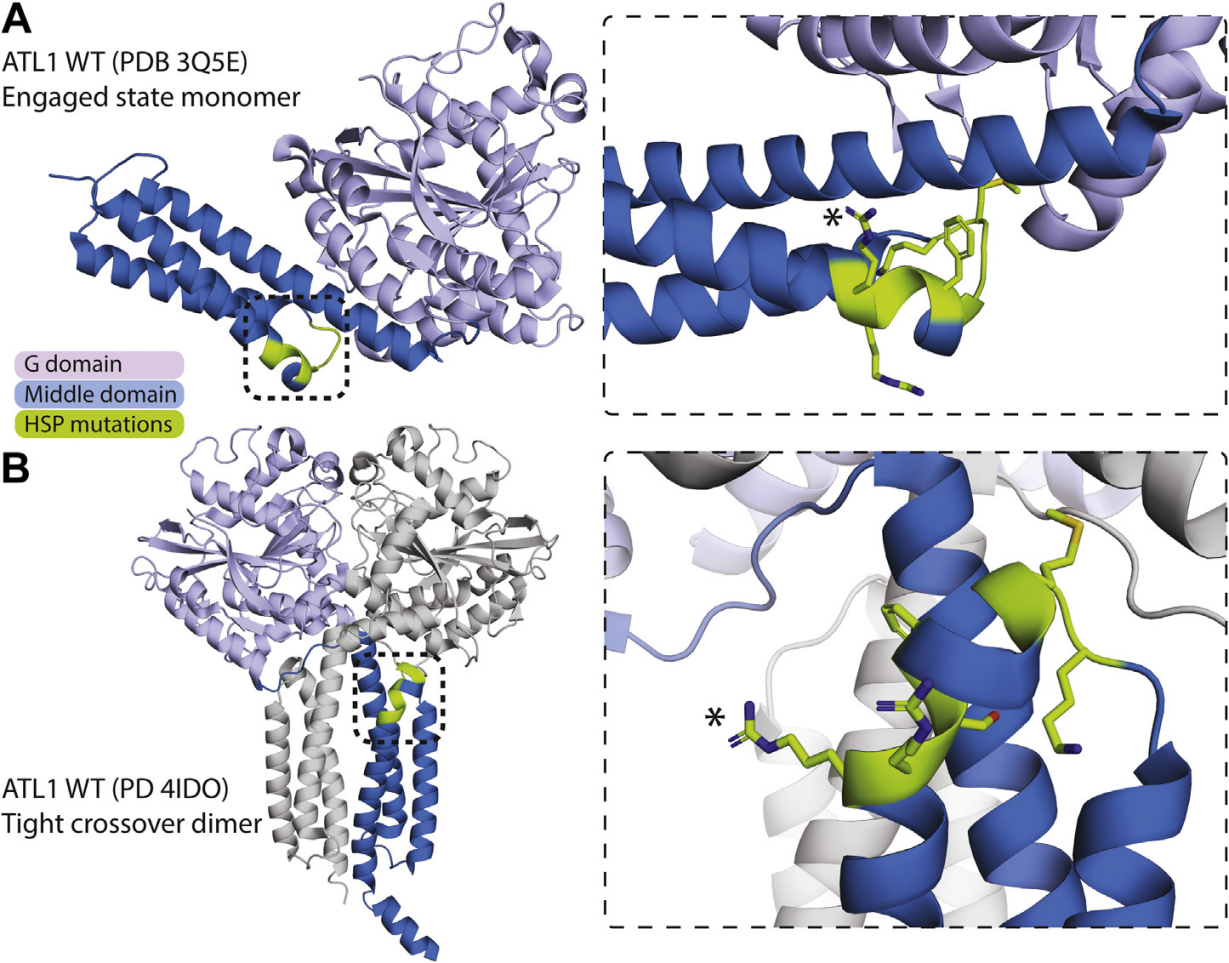
**Cluster of SPG3A HSP-causative mutations proximal to the N416 insertion.***A*, localization of an apparent mutational hotspot demarcated on the structure of ATL1 WT in the prehydrolysis, engaged state (PDB 3Q5E). HSP-associated, mutated residues shown in *green* and colored by atom on the *right*. *Asterisk* (∗) indicates R416, which directly precedes the insertion mutation described here. *Black boxes* indicate boundaries of zoom-in view on the *right* in (*A* and *B*). *B*, localization of the cluster in the structure of the tight crossover dimer bound to GDP•AlF_4_^−^ (PDB 4IDO; (25)). Coloring as in (*A*) for chain B and chain A is shown in *gray*.

Interestingly, there is a cluster of HSP-associated mutations surrounding the N417ins mutation studied here, ranging from residues K407 to R416 (Table 2). Figure 7 illustrates the localization of this cluster in both the prehydrolysis (form 2) and the tight crossover dimer (form 3) between α-helix 2 and 3 of the middle domain, and adjacent to the linker region between the G and middle domains. While the molecular mechanisms of these mutations have not been characterized, there is a general clinical trend of early onset of symptoms and autosomal dominant inheritance. The only report of recombinant expression for any of these mutations (F413L and R415W) found them to be insoluble, likely explaining their pathogenesis (23). Additionally, while a majority of *SPG3A* HSP-causing mutations result in pure cases (especially with autosomal dominant cases) (3, 13, 59), many of the mutations in this cluster are found in complex cases including the case we present here. While we do not anticipate the pathogenic mechanism of every mutation within this cluster to be the same or similar, it is evident that this region of the middle domain is crucial in maintaining proper ATL1 function. The variability observed across the cases within this cluster points toward a more complex physiological mechanism beyond what enzyme kinetics alone would likely explain, for example, involving a potential gain-of-function mechanism on the level of the protein’s cellular role, in this case membrane tethering and fusion.

**Table 2:** HSP-causative *SPG3A* mutations clustered in the middle domain near the N417ins mutation

Mutation	Pure or complex case(s)	Inheritance	Early *versus* late onset	References
K407R	Pure	AD	Early	(78)
M408V	Complex and pure	AD	Early	(71)
M408T	Complex	AD	Early	(79)
G409D	Complex	S	Early	(53, 70)
G410R	Complex	AD	(Mostly) early	(80)
F413V	Complex	AD	Early	(81)
F413L∗	Pure	AD	Early	(82)
S414R	Not described	AD	Early	(83)
R415W∗	Pure (one complex)	AD	(Mostly) early	(84, 85, 86, 87)
R415Q	Pure	AD	Early	(87)
R416C	Complex and pure	AD	(Mostly) late	(65, 88)
R416H	Complex	AD	Early and late	(89)
N417ins	Complex		Early	This study.

All mutations within this cluster are caused by missense mutations and result in single amino acid replacements, indicated in the first column. Subsequent columns detail whether the reported case(s) are pure or complex, the mode of inheritance (autosomal dominant = AD, sporadic = S), early *versus* late symptom onset (early is defined as <10 years old), and references. Mutants with an asterisk (∗) are insoluble when expressed recombinantly as the catalytic core (23).

While the HSP-associated mutation presented here demonstrates a novel gain-of-function disease mechanism in ATL1, a recent study characterized two gain-of-function mutations in ATL3 (Y192C and P338R) that had been identified in hereditary sensory neuropathy patients (38, 39, 60). Like the novel ATL1 insertion mutation reported here, both ATL3 disease-associated variants show unaltered GTPase activity and increased membrane tethering rates; however, in contrast to ATL1 N417ins, the propensity for nucleotide-dependent dimerization of the ATL3 mutants appears more severely reduced (39). In several mammalian cells models, including HSN patient fibroblasts and murine cortical neurons, expression of the ATL3 Y192C and P338R variants caused a striking phenotype characterized by lateral bundling of ER tubules caused by excessive tethering, with a partial collapse of the reticular ER caused by significant membrane fusion defects. Unlike the model proposed for the ATL3 HSN-associated mutants, which suggests a failure in establishing nucleotide-dependent ATL crossover dimers required for fusion, ATL1 N417ins was shown to be able to adopt this conformation similar to the wild-type protein, with the mutation likely affecting a pre-GTP hydrolysis step. Notably, both Y192 and P338 in ATL3 are proximal to the intramolecular G-middle domain interface of the engaged state that we hypothesize is disrupted in the ATL1 N417ins mutation, potentially suggesting a common pathogenic mechanism. Mutations at residues corresponding to Y192 and P338 of ATL3 in ATL1 (Y196 and P342, respectively) have been identified in HSP patients (61, 62). In addition, the conserved P342 along with P344 has been shown to play a pivotal role in rigid body rotation between the G and middle domain in ATL (23) and the bundle signaling element in dynamin (63). The parallels between the behavior of ATL1 N417ins and ATL3 Y192C and P338R mutations support our proposed mechanism and highlight the critical role the G-middle domain interface plays in ATL fusion regulation.

Another notable feature of the case described in this study is the presence of a thinning corpus callosum (TCC; Fig. 1*A*), which has been described as a subcategory of complex HSP, most commonly attributed to autosomal recessive mutations in *SPG11* (spatacsin), with fewer cases caused by *SPG21* (maspardin), *SPG32*, *SPG15*, and *TUBB4A* (13, 64). A more recent study identified a family in South Africa with three generations of late-onset AD-HSP with a pattern of TCC stemming from the *SPG3A* R416C mutation ((65); Table 2 and Fig. 7). Although the cases in the study by Orlacchio *et al.* and a majority of HSP-TCC cases display some level of cognitive impairment, the proband in the present study has normal cognitive functioning.

As is the case in some complex HSP cases, the proband here has shown features of epilepsy as established by an EEG. Recently, significantly decreased ATL1 expression levels have been identified in the temporal lobe neocortex of patients with temporal lobe epilepsy (TLE) (66). Upon overexpression of ATL1 in an epileptic mouse model, seizure occurrence decreased resulting from inhibition of spontaneous action potential. While the exact mechanism remains elusive, a link may be drawn in *SPG3A* HSP patients who experience epileptic symptoms.

A frequent theme with HSP patients is an early diagnosis of cerebral palsy (CP), as seen also in the case reported here. Because HSPs are very rare and genetically heterogeneous disorders, they can be difficult to clinically identify and diagnose, especially if there is no family history. Since CP usually manifests at the beginning of life, it is not uncommon for patients with early onset HSP and spastic di- or quadriplegia to first be diagnosed with CP (67). Since ATL1 is the most frequently mutated in early onset cases, it is not surprising that many of these patients first receive a CP diagnosis, especially if disease progression is slow, since CP is considered a static condition (68, 69, 70, 71, 72, 73). Differentiating between the two conditions based on gait patterns is one potential diagnostic tool (74). A definitive diagnosis relies on genetic testing, and even then, pathogenic mechanisms can vary drastically requiring a detailed analysis of the underlying molecular mechanisms, as exemplified here.

## Experimental procedures

### Whole exome sequencing

The proband and both parents were consented and enrolled into the research study WIRB Protocol #20120789. The patient was 14 years of age at the time of enrollment and verbal assent was obtained. Whole exome sequencing was performed on all three, and an annotated file containing variants in the family members was filtered to include novel, private, or rare variants according to the Genome Aggregation Database (https://gnomad.broadinstitute.org/). Mode of inheritance and disease association, followed by detailed analyst assessment for genotype–phenotype correlation, disease mechanism, and literature review were performed. Variants predicted to be damaging by multiple tools: Combined Annotation Dependent Depletion (https://cadd.gs.washington.edu/), ExAC’s probability of loss-of-function intolerance score and missense z-score (pLi, z-score) and Genomic Evolutionary Rate Profiling (GERP, http://mendel.stanford.edu/SidowLab/downloads/gerp/) algorithms were considered as candidate genes responsible for the proband’s phenotype.

### Protein expression and purification

The catalytic core of human ATL1 WT (residues 1–449) and N417ins (residues 1–447 for X-ray crystallography; residues 1–450 for enzyme assays) were each cloned into a pET21 vector using standard molecular cloning methods. Each construct had a C-terminal HIS_10_ tag, excect the crystallography construct that had a HIS_6_ tag. Protein was overexpressed in *E. coli* BL21(DE3) grown in Terrific Broth to an OD_600_ ∼0.8, followed by induction for 16 h at 18 °C with 0.5 mM IPTG. Cells were harvested at 4500*g* and resuspended in Ni^2+^-NTA buffer A (25 mM Tris-HCl pH 8.5, 500 mM NaCl, 20 mM imidazole), flash frozen in liquid nitrogen, and stored at −80 °C. For purification, cell pellets were thawed at RT, sonicated, and spun at 39,000*g* to clear insoluble material. Supernatant was loaded onto Ni^2+^-NTA resin (Qiagen) equilibrated in buffer A at a ratio of 1 ml resin: 1 l culture. Loaded resin was washed with 15 column volumes (CV) of buffer A, then eluted with 3 CV Ni^2+^-NTA buffer B (25 mM Tris-HCl pH 8.5, 500 mM NaCl, 500 mM imidazole). Elution was buffer exchanged into desalt buffer (25 mM Tris-HCl pH 7.5, 400 mM NaCl, 5 mM EDTA) using a HiPrep 26/10 desalting column (GE Life Sciences). Concentrated elution was run on a GE S200 16/60 equilibrated with gel filtration buffer (25 mM Tris-HCl pH 7.5, 100 mM NaCl). Peak fractions were analyzed *via* SDS-PAGE. Target protein-containing fractions were concentrated, frozen in liquid nitrogen, and stored at −80 °C.

The G domain (residues 1–339 of human ATL1) was purified as above with the following exceptions. The construct was cloned into a pET28-based vector with an N-terminal HIS_6_ tag followed by a SUMO tag and a Ulp1 cleavage site. After buffer exchange into desalt buffer (without EDTA), removal of the HIS_6_-SUMO tag was carried out on ice overnight with Ulp1 protease. The protease and affinity tag were removed with a 5 ml HisTrap HP column (GE), and flowthrough was collected, concentrated, and loaded onto a GE S200 16/60 gel filtration column as above.

### X-ray crystallography data collection and structure determination

hATL1 N417ins (residues 1–447, with C-terminal His_6_ tag) was crystallized using the hanging drop vapor diffusion method. Protein at 20 and 30 mg/ml was first incubated with 4 mM MgCl_2_, 2 mM GDP, 2 mM AlCl_2_, and 20 mM NaF on ice for 1 h. Reservoir buffer containing 0.1 M Tris-HCl pH 8.1, 2% Tacsimate pH 8, 18% PEG 3350 was combined with protein mixture at 1:1 ratio on a glass cover slip before sealing and incubating at 20 °C. Prior to freezing, crystals were soaked in a cryoprotectant solution containing crystallization buffer with 25% glycerol for ∼5 min. X-ray diffraction data were collected at the Cornell High Energy Synchrotron Source (CHESS).

Phase determination was carried out with molecular replacement using PHENIX (75) and coordinates of the WT ATL1 catalytic core (residues 1–446) bound to GDP•AlF_4_^−^ (PDB 4IDO; (25)) as the search model. The N417ins mutant structure was built and refined in PHENIX (75) and Coot (76). Statistics for data collection and refinement are summarized in Table 1.

### Phosphate-release kinetics

An Enzchek phosphate kit (Thermo Fisher Scientific) was used to measure apparent catalytic efficiencies for the catalytic-core ATL1 WT and N417ins mutant proteins. Reactions were set up according to the manufacturer’s instructions but were scaled to 200 μl and run in the presence of reaction buffer (25 mM Tris-HCl pH 7.5, 100 mM NaCl, and 2 mM MgCl_2_) with protein concentrations ranging from 0 to 2 μM and GTP concentration constant at 500 μM. Over a 45 min reaction period, accumulation of P_i_ was measured as a spectrophotometric shift from 330 nm to 360 nm as 2-amino-6-mercapto-7-methylpurine riboside (MESG) was converted to ribose-1-phosphate and 2-amino-6-mercapto-7-methylpurine by purine nucleoside phosphorylase. Absorbance values were converted to P_i_ concentrations based on a standard curve. Turnover efficiencies (*k*_*cat*_) were calculated using three technical replicates and two biological replicates for each protein.

### Size-exclusion chromatography coupled to multiangle light scattering

ATL1 WT and N417ins catalytic-core constructs used for kinetic experiments were loaded at 50 μM onto a Superdex Increase 200 10/300 GL column (GE) equilibrated in 25 mM Tris-HCl pH 7.5, 100 mM NaCl, 2 mM EGTA, 4 mM MgCl_2_. All samples were preincubated with 2 mM GDP, 2 mM GppNHp, 2 mM GDP in the presence of 2 mM AlCl_2,_ 20 mM NaF, and 4 mM EGTA, or without nucleotide. As protein eluted from the column, it was passed through a static 18-angle light scattering detection unit (DAWN HELEOS-II) and a refractive index detector (Optilab T-rEX), both of which record signal intensity in volts. Wyatt’s Astra VI software was used to determine both molecular weight and mass distribution through each sample experiment. Monomeric BSA (Sigma) was used to normalize signal measured by light scattering detectors (49).

### Liposome preparation

All lipids were purchased from Avanti Polar Lipids either reconstituted in chloroform or as desiccate, which were reconstituted after purchase. For all experiments, lipids were prepared as 10 mM stocks at a molar ratio of 1% 18:1 DGS-NTA(Ni) to 99% 18:1 (Δ9-*cis*) PC. Appropriate volumes of chloroform stocks were transferred to glass tubes and chloroform was evaporated under a N_2_ air stream for 15 to 20 min followed by desiccation for at least 1.5 h to remove remaining solvent. Lipids were reconstituted in 25 mM Tris-HCl pH7.5, 100 mM NaCl and vortexed intermittently for 30 min to create multilamellar vesicles (MLV). MLVs were then subjected to ten freeze–thaw cycles between liquid N_2_ and a room temperature water bath to reduce the multilamellar structures in favor of unilamellar vesicles. Lipids were stored at −80 °C until needed. Upon thawing, lipids were extruded 21 times through a filter with 100 nm pore size to achieve a relatively homogenous size solution of unilamellar vesicles. Vesicles were used immediately.

### Liposome tethering assay

His_10_-tagged ATL1 WT or N417ins protein was loaded onto Ni^2+^-modified vesicles as a 2× reaction stock with 2 mM lipid vesicles (1 mM final) and 0.5 to 3 μM protein (0.25 μM–1.5 μM final) by incubating at room temperature for 30 min in the presence of reaction buffer (25 mM Tris-HCl pH 7.5, 100 mM NaCl, 4 mM MgCl_2_). Vesicle tethering was measured spectrophotometrically at OD_360_ for 45 min upon dilution of reaction stock with reaction buffer ±500 μM GTP. Images of aggregate vesicles were taken on a BioRad Chemidoc imaging system. All reactions were carried out in triplicate with two biological replicates and data processing was done in GraphPad Prism. Signal for the three “minus GTP” reactions per condition were averaged and subtracted from each “plus GTP” reaction. To determine apparent tethering rates, data were excluded after signal plateau was reached and before signal drop-off (if there was no terminal signal drop, data were not excluded), and the single-phase association rate was calculated and averaged across replicates. The rates were plotted, and the regression for the second linear region was calculated to determine the rate constant for each protein. Statistical significance was determined using an unpaired *t* test.

### Liposome flotation assay

Liposome flotation assays were conducted using a Nycodenz concentration gradient, as detailed in Liu *et al.*, 2015 (26). Nycodenz powder was dissolved in vesicle tethering reaction buffer at 25% and 70% stock concentrations. Protein-loaded vesicles, prepared as described above, with 2 μM protein and 2 mM lipids, were combined at a 1:1 ratio with the 70% Nycodenz stock, for a final 35% Nycodenz solution (final concentrations of 1 μM protein and 1 mM lipids). The concentration gradient was set up with the bottom 20% of the total volume consisting of the 35% Nycodenz and loaded vesicle solution, the middle 70% consisting of the 25% Nycodenz solution, and the top 10% of the volume being vesicle tethering reaction buffer. These gradients were subjected to ultracentrifugation at 200,310*g* for 2 h at 4 °C, then fractions were taken from the top, middle, and bottom of each gradient and analyzed *via* SDS-PAGE and stained with SYPRO Ruby according to manufacturer’s instructions.

### Western blotting

Human ATL1 WT (residues 1–558) or ATL1 N417ins (residues 1–559) were cloned into a pcDNA4 vector containing a C-terminal c-myc affinity tag using standard molecular cloning methods. These were used to transiently transfect NIH 3T3 WT and NIH 3T3 ATL1/2/3 TKO cells using Avalanche-Omni (EZ Biosystems) according to manufacturer’s recommendations. Cells were washed in cold PBS three times and then harvested by scraping and lysed in 25 mM Tris-HCl pH 7.5, 100 mM NaCl, 1% Triton X-100. Total protein in lysates was determined by Bradford reagent (Bio-Rad) and normalized before loading. Gels were run for 1 h at 210 V, then transferred to a PVDF membrane. Blots were blocked either 1.5 h at room temperature or overnight at 4 °C in SuperBlock TBS blocking buffer (ThermoFisher Scientific), followed by incubation with 1:1000 mouse α-c-myc monoclonal 9E10 antibody (Abcam) or 1:60,000 rabbit α-calnexin (Abcam) in Tris-buffered saline with 0.1% Tween-20 (TBST) for 1 h at room temperature, followed by a 30-min incubation with 1:5000 goat α-mouse-HRP polyclonal antibody or 1:5000 goat α-rabbit-HRP polyclonal antibody (Thermo Fisher Scientific) diluted in TBST. Each incubation period was proceeded by 3 × 5-min washes with TBST. Blots were developed using an enhanced chemiluminescent HRP substrate and imaged with a BioRad Chemidoc imaging system.

### Mammalian cell immunofluorescence and imaging

Mammalian expression vectors containing ATL1 WT or N417ins mutant protein were transfected into NIH 3T3 WT and NIH 3T3 ATL1/2/3 TKO cells using Avalanche-Omni (EZ Biosystems) as described above or cotransfected with ^mCherry^SEC61B into U2OS cells using polyethylenimine (PEI). Twenty-four hours after transfections, cells were processed. Cells were washed three times with PBS between each step. They were first fixed for 20 min with 4% formaldehyde followed by permeabilization with 0.1% Triton X-100 for 10 min and then blocked in 10% BSA for 1.5 h. Cells were then incubated for 1.5 h with a 1:400 dilution of mouse α-c-myc monoclonal 9E10 antibody (Abcam) in 5% BSA, then finally in 1:400 α-mouse AlexaFluor-488 in 5% BSA for 1 h. Cells were washed a final time and then treated with ProLong Gold Antifade mountant (Thermo Fisher Scientific) before sealing with a coverslip. Images were taken on a Perkins-Elmer UltraView spinning disc confocal microscope with a Nikon Plan Apo 60xA/1.4 oil objective. For each condition, a minimum of 70 cells were imaged for quantification across three experimental replicates (except for ATL1 WT in NIH 3T3 ATL1/2/3 TKO cells, for which 46 cells were imaged). Image brightness and contrast were adjusted using ImageJ (77) and then visually assessed for the presence of puncta and tubules. For each category, the percent of total cells was averaged across replicates, and then statistical significance was determined by one-way ANOVA.

### Limited proteolysis assay

A 2× stock of each the ATL1 WT and N417ins catalytic core proteins and the WT G domain were prepared in gel filtration buffer (with 4 mM MgCl_2_ added) at 4 μM. Each was incubated with corresponding nucleotides (2 mM GDP or 2 mM GDP, 2 mM AlCl_2_, 20 mM NaF, and 4 mM EGTA) for 30 min at RT and then placed on ice. Proteinase K (New England Biolabs) was also prepared as 2× stocks at 2 μg/ml and 20 μg/ml in gel filtration buffer and kept on ice. ATL1 samples were combined with PK stocks at 1:1 ratio, and reactions were carried out on ice for 15 min, then quenched with 2 mM PMSF in DMSO and 5× SDS loading buffer. Samples were boiled immediately and analyzed with SDS-PAGE gels. Gels were stained with Coomassie Brilliant Blue.

### Fluorescent-dye labeling

To achieve site specificity of protein labeling with maleimide conjugated dyes, a surface-exposed cysteine was mutated to alanine (C375A) and a single cysteine was introduced either on the G domain (K296C) or middle domain (K400C). When labeled, these combinations of mutations were shown to singly and efficiently modify ATL1 without affecting protein function (25, 44). For all FRET labeling reactions, catalytic cores of ATL1 WT or N417ins mutant protein (both with C-terminal His_6_ tags) were used with the mutations indicated above. Each reaction contained 100 μM protein and 150 μM maleimide-conjugated AlexaFluor-488, AlexaFluor-647, or both (Thermo Fisher Scientific) in FRET labeling buffer (25 mM HEPES-NaOH pH7, 100 mM NaCl). Reactions were incubated on ice for 30 min and then excess dye was removed with a NAP-5 column (GE) equilibrated with FRET reaction buffer (25 mM HEPES-KOH pH 7.5, 100 mM NaCl, 2 mM MgCl_2_). Final protein and dye concentrations were determined spectrophotometrically using a Nanodrop spectrophotometer (ThermoFisher Scientific).

### Equilibrium inter- and intramolecular FRET measurements

All steady-state intermolecular FRET experiments were carried out at a concentration of 5 μM total protein with a 1:1 M ratio of donor to acceptor-labeled protein (2.5 μM donor: 2.5 μM acceptor) in FRET reaction buffer, preincubated with 2 mM GDP, 2 mM GDP in the presence of 2 mM AlCl_2,_ 20 mM NaF, and 4 mM EGTA, or without nucleotide at room temperature for 30 min. Reactions were measured using a Gemini EM microplate reader (Molecular Devices) at an excitation wavelength of 473 nm and emission spectra between 495 and 745 nm in 10 nm steps. iFRET equilibrium measurements were carried out with the same parameters, except that reactions contained a total of 5 μM protein with a 1:10 ratio of labeled to unlabeled protein (0.5 μM dually labeled and 4.5 μM unlabeled protein). GraphPad Prism was used for all data analysis, with FRET efficiencies calculated with the ratio *I*_*acceptor*_/(*I*_*donor*_ + *I*_*acceptor*_) where *I* was the signal intensity at peak emission wavelengths (515 nm and 655 nm for the donor and acceptor, respectively). All reactions were carried out in triplicate with two biological replicates. One-way ANOVA tests were carried out to determine statistically significant changes in FRET efficiencies.

## Data availability

The atomic coordinates and structure factors have been deposited in the Protein Data Bank, www.rcsb.org (PDB ID codes 7OL3). All other data used for the study are presented or cited in the article or the supporting information.

## Supporting information

This article contains supporting information (77).

## Conflict of interest

The authors declare that there are no conflicts of interests with the contents of this article.

## References

[1] DeLuca G.C., Ebers G.C., Esiri M.M. (2004). The extent of axonal loss in the long tracts in hereditary spastic paraplegia. Neuropathol. Appl. Neurobiol..

[2] Fink J.K. (2013). Hereditary spastic paraplegia: Clinico-pathologic features and emerging molecular mechanisms. Acta Neuropathol..

[3] Fink J.K. (2006). Hereditary spastic paraplegia. Curr. Neurol. Neurosci. Rep..

[4] Blackstone C. (2012). Cellular pathways of hereditary spastic paraplegia. Annu. Rev. Neurosci..

[5] Blackstone C. (2020). Early-onset hereditary spastic paraplegia: The possibility of a genetic diagnosis. Dev. Med. Child Neurol..

[6] Fink J.K., Rosenberg R.N., Pascual J.M. (2020). Rosenberg’s Molecular and Genetic Basis of Neurological and Psychiatric Disease: Volume 1.

[7] Blackstone C. (2018). Hereditary spastic paraplegia. Handb. Clin. Neurol..

[8] Willkomm L., Heredia R., Hoffmann K., Wang H., Voit T., Hoffman E.P., Cirak S. (2016). Homozygous mutation in atlastin GTPase 1 causes recessive hereditary spastic paraplegia. J. Hum. Genet..

[9] Klebe S., Stevanin G., Depienne C. (2015). Clinical and genetic heterogeneity in hereditary spastic paraplegias: From SPG1 to SPG72 and still counting. Rev. Neurol. (Paris).

[10] Zhao X., Alvarado D., Rainier S., Lemons R., Hedera P., Weber C.H., Tukel T., Apak M., Heiman-Patterson T., Ming L., Bui M., Fink J.K. (2001). Mutations in a newly identified GTPase gene cause autosomal dominant hereditary spastic paraplegia. Nat. Genet..

[11] Abel A., Fonknechten N., Hofer A., Dürr A., Cruaud C., Voit T., Weissenbach J., Brice A., Klimpe S., Auburger G., Hazan J. (2004). Early onset autosomal dominant spastic paraplegia caused by novel mutations in SPG3A. Neurogenetics.

[12] Namekawa M., Ribai P., Nelson I., Forlani S., Fellmann F., Goizet C., Depienne C., Stevanin G., Ruberg M., Dürr A., Brice A. (2006). SPG3A is the most frequent cause of hereditary spastic paraplegia with onset before age 10 years. Neurology.

[13] Depienne C., Stevanin G., Brice A., Durr A. (2007). Hereditary spastic paraplegias: An update. Curr. Opin. Neurol..

[14] Praefcke G.J., McMahon H.T. (2004). The dynamin superfamily: Universal membrane tubulation and fission molecules?. Nat. Rev. Mol. Cell Biol..

[15] Tuma P.L., Collins C.A. (1994). Activation of dynamin GTPase is a result of positive cooperativity. J. Biol. Chem..

[16] Gasper R., Meyer S., Gotthardt K., Sirajuddin M., Wittinghofer A. (2009). It takes two to tango: Regulation of G proteins by dimerization. Nat. Rev. Mol. Cell Biol..

[17] Ford M.G.J., Chappie J.S. (2019). The structural biology of the dynamin-related proteins: New insights into a diverse, multitalented family. Traffic.

[18] Orso G., Pendin D., Liu S., Tosetto J., Moss T.J., Faust J.E., Micaroni M., Egorova A., Martinuzzi A., McNew J.A., Daga A. (2009). Homotypic fusion of ER membranes requires the dynamin-like GTPase atlastin. Nature.

[19] Hu J., Shibata Y., Zhu P.P., Voss C., Rismanchi N., Prinz W.A., Rapoport T.A., Blackstone C. (2009). A class of dynamin-like GTPases involved in the generation of the tubular ER network. Cell.

[20] Betancourt-Solis M.A., Desai T., McNew J.A. (2018). The atlastin membrane anchor forms an intramembrane hairpin that does not span the phospholipid bilayer. J. Biol. Chem..

[21] Liu T.Y., Bian X., Sun S., Hu X., Klemm R.W., Prinz W.A., Rapoport T.A., Hu J. (2012). Lipid interaction of the C terminus and association of the transmembrane segments facilitate atlastin-mediated homotypic endoplasmic reticulum fusion. Proc. Natl. Acad. Sci. U. S. A..

[22] Faust J.E., Desai T., Verma A., Ulengin I., Sun T.L., Moss T.J., Betancourt-Solis M.A., Huang H.W., Lee T., McNew J.A. (2015). The atlastin C-terminal tail is an amphipathic helix that perturbs the bilayer structure during endoplasmic reticulum homotypic fusion. J. Biol. Chem..

[23] Byrnes L.J., Sondermann H. (2011). Structural basis for the nucleotide-dependent dimerization of the large G protein atlastin-1/SPG3A. Proc. Natl. Acad. Sci. U. S. A..

[24] Bian X., Klemm R.W., Liu T.Y., Zhang M., Sun S., Sui X., Liu X., Rapoport T.A., Hu J. (2011). Structures of the atlastin GTPase provide insight into homotypic fusion of endoplasmic reticulum membranes. Proc. Natl. Acad. Sci. U. S. A..

[25] Byrnes L.J., Singh A., Szeto K., Benvin N.M., O'Donnell J.P., Zipfel W.R., Sondermann H. (2013). Structural basis for conformational switching and GTP loading of the large G protein atlastin. EMBO J..

[26] Liu T.Y., Bian X., Romano F.B., Shemesh T., Rapoport T.A., Hu J. (2015). Cis and trans interactions between atlastin molecules during membrane fusion. Proc. Natl. Acad. Sci. U. S. A..

[27] Powers R.E., Wang S., Liu T.Y., Rapoport T.A. (2017). Reconstitution of the tubular endoplasmic reticulum network with purified components. Nature.

[28] Wu F., Hu X., Bian X., Liu X., Hu J. (2015). Comparison of human and Drosophila atlastin GTPases. Protein Cell.

[29] Moss T.J., Andreazza C., Verma A., Daga A., McNew J.A. (2011). Membrane fusion by the GTPase atlastin requires a conserved C-terminal cytoplasmic tail and dimerization through the middle domain. Proc. Natl. Acad. Sci. U. S. A..

[30] Hu X., Wu F., Sun S., Yu W., Hu J. (2015). Human atlastin GTPases mediate differentiated fusion of endoplasmic reticulum membranes. Protein Cell.

[31] Rismanchi N., Soderblom C., Stadler J., Zhu P.P., Blackstone C. (2008). Atlastin GTPases are required for Golgi apparatus and ER morphogenesis. Hum. Mol. Genet..

[32] Ulengin I., Park J.J., Lee T.H. (2015). ER network formation and membrane fusion by atlastin1/SPG3A disease variants. Mol. Biol. Cell.

[33] O’Donnell J.P., Byrnes L.J., Cooley R.B., Sondermann H. (2018). A hereditary spastic paraplegia–associated atlastin variant exhibits defective allosteric coupling in the catalytic core. J. Biol. Chem..

[34] Montagna A., Vajente N., Pendin D., Daga A. (2020). *In vivo* analysis of CRISPR/Cas9 induced atlastin pathological mutations in Drosophila. Front. Neurosci..

[35] Liu X., Guo X., Niu L., Li X., Sun F., Hu J., Wang X., Shen K. (2019). Atlastin-1 regulates morphology and function of endoplasmic reticulum in dendrites. Nat. Commun..

[36] Zhao G., Zhu P.P., Renvoisé B., Maldonado-Báez L., Park S.H., Blackstone C. (2016). Mammalian knock out cells reveal prominent roles for atlastin GTPases in ER network morphology. Exp. Cell Res..

[37] Guelly C., Zhu P.P., Leonardis L., Papić L., Zidar J., Schabhüttl M., Strohmaier H., Weis J., Strom T.M., Baets J., Willems J., De Jonghe P., Reilly M.M., Fröhlich E., Hatz M. (2011). Targeted high-throughput sequencing identifies mutations in atlastin-1 as a cause of hereditary sensory neuropathy type I. Am. J. Hum. Genet..

[38] Fischer D., Schabhüttl M., Wieland T., Windhager R., Strom T.M., Auer-Grumbach M. (2014). A novel missense mutation confirms ATL3 as a gene for hereditary sensory neuropathy type 1. Brain.

[39] Krols M., Detry S., Asselbergh B., Almeida-Souza L., Kremer A., Lippens S., De Rycke R., De Winter V., Müller F.J., Kurth I., McMahon H.T., Savvides S.N., Timmerman V., Janssens S. (2018). Sensory-neuropathy-causing mutations in ATL3 cause aberrant ER membrane tethering. Cell Rep..

[40] Karczewski K.J., Francioli L.C., Tiao G., Cummings B.B., Alföldi J., Wang Q., Collins R.L., Laricchia K.M., Ganna A., Birnbaum D.P., Gauthier L.D., Brand H., Solomonson M., Watts N.A., Rhodes D. (2020). The mutational constraint spectrum quantified from variation in 141,456 humans. Nature.

[41] Landrum M.J., Lee J.M., Riley G.R., Jang W., Rubinstein W.S., Church D.M., Maglott D.R. (2014). ClinVar: Public archive of relationships among sequence variation and human phenotype. Nucleic Acids Res..

[42] Namekawa M., Muriel M.P., Janer A., Latouche M., Dauphin A., Debeir T., Martin E., Duyckaerts C., Prigent A., Depienne C., Sittler A., Brice A., Ruberg M. (2007). Mutations in the SPG3A gene encoding the GTPase atlastin interfere with vesicle trafficking in the ER/Golgi interface and Golgi morphogenesis. Mol. Cell. Neurosci..

[43] Wang S., Romano F.B., Field C.M., Mitchison T.J., Rapoport T.A. (2013). Multiple mechanisms determine ER network morphology during the cell cycle in Xenopus egg extracts. J. Cell Biol..

[44] O'Donnell J.P., Cooley R.B., Kelly C.M., Miller K., Andersen O.S., Rusinova R., Sondermann H. (2017). Timing and reset mechanism of GTP hydrolysis-driven conformational changes of atlastin. Structure.

[45] Pendin D., Tosetto J., Moss T.J., Andreazza C., Moro S., McNew J.A., Daga A. (2011). GTP-dependent packing of a three-helix bundle is required for atlastin-mediated fusion. Proc. Natl. Acad. Sci. U. S. A..

[46] Yan L., Sun S., Wang W., Shi J., Hu X., Wang S., Su D., Rao Z., Hu J., Lou Z. (2015). Structures of the yeast dynamin-like GTPase Sey1p provide insight into homotypic ER fusion. J. Cell Biol..

[47] Cao Y.L., Meng S., Chen Y., Feng J.X., Gu D.D., Yu B., Li Y.J., Yang J.Y., Liao S., Chan D.C., Gao S. (2017). MFN1 structures reveal nucleotide-triggered dimerization critical for mitochondrial fusion. Nature.

[48] Qi Y., Yan L., Yu C., Guo X., Zhou X., Hu X., Huang X., Rao Z., Lou Z., Hu J. (2016). Structures of human mitofusin 1 provide insight into mitochondrial tethering. J. Cell Biol..

[49] O’Donnell J.P., Kelly C.M., Sondermann H. (2020). Dynamin Superfamily GTPases 2020.

[50] Touw W.G., Baakman C., Black J., Te Beek T.A., Krieger E., Joosten R.P., Vriend G. (2015). A series of PDB-related databanks for everyday needs. Nucleic Acids Res..

[51] Kabsch W., Sander C. (1983). Dictionary of protein secondary structure: Pattern recognition of hydrogen-bonded and geometrical features. Biopolymers.

[52] Wang N., Rapoport T.A. (2019). Reconstituting the reticular ER network - mechanistic implications and open questions. J. Cell Sci..

[53] Zhao G.H., Liu X.M. (2017). Clinical features and genotype-phenotype correlation analysis in patients with ATL1 mutations: A literature reanalysis. Transl. Neurodegener..

[54] Xiao X.W., Du J., Jiao B., Liao X.X., Zhou L., Liu X.X., Yuan Z.H., Guo L.N., Wang X., Shen L., Lin Z.Y. (2019). Novel ATL1 mutation in a Chinese family with hereditary spastic paraplegia: A case report and review of literature. World J. Clin. Cases.

[55] Botzolakis E.J., Zhao J., Gurba K.N., Macdonald R.L., Hedera P. (2011). The effect of HSP-causing mutations in SPG3A and NIPA1 on the assembly, trafficking, and interaction between atlastin-1 and NIPA1. Mol. Cell. Neurosci..

[56] Meijer I.A., Dion P., Laurent S., Dupré N., Brais B., Levert A., Puymirat J., Rioux M.F., Sylvain M., Zhu P.P., Soderblom C., Stadler J., Blackstone C., Rouleau G.A. (2007). Characterization of a novel SPG3A deletion in a French-Canadian family. Ann. Neurol..

[57] Cooley R.B., Arp D.J., Karplus P.A. (2010). Evolutionary origin of a secondary structure: π-Helices as cryptic but widespread insertional variations of α-helices that enhance protein functionality. J. Mol. Biol..

[58] Nixon-Abell J., Obara C.J., Weigel A.V., Li D., Legant W.R., Xu C.S., Pasolli H.A., Harvey K., Hess H.F., Betzig E., Blackstone C., Lippincott-Schwartz J. (2016). Increased spatiotemporal resolution reveals highly dynamic dense tubular matrices in the peripheral ER. Science.

[59] Battini R., Fogli A., Borghetti D., Michelucci A., Perazza S., Baldinotti F., Conidi M.E., Ferreri M.I., Simi P., Cioni G. (2011). Clinical and genetic findings in a series of Italian children with pure hereditary spastic paraplegia. Eur. J. Neurol..

[60] Kornak U., Mademan I., Schinke M., Voigt M., Krawitz P., Hecht J., Barvencik F., Schinke T., Gießelmann S., Beil F.T., Pou-Serradell A., Vílchez J.J., Beetz C., Deconinck T., Timmerman V. (2014). Sensory neuropathy with bone destruction due to a mutation in the membrane-shaping atlastin GTPase 3. Brain.

[61] McCorquodale D.S., Ozomaro U., Huang J., Montenegro G., Kushman A., Citrigno L., Price J., Speziani F., Pericak-Vance M.A., Züchner S. (2011). Mutation screening of spastin, atlastin, and REEP1 in hereditary spastic paraplegia. Clin. Genet..

[62] De Bot S.T., Veldink J.H., Vermeer S., Mensenkamp A.R., Brugman F., Scheffer H., van den Berg L.H., Kremer H.P., Kamsteeg E.J., van de Warrenburg B.P. (2013). ATL1 and REEP1 mutations in hereditary and sporadic upper motor neuron syndromes. J. Neurol..

[63] Chappie J.S., Acharya S., Leonard M., Schmid S.L., Dyda F. (2010). G domain dimerization controls dynamin's assembly-stimulated GTPase activity. Nature.

[64] Lamartine S Monteiro M., Vandernoot I., Desmyter L., Wermenbol V., Naeije G., Remiche G. (2020). Corpus callosum thinning in autosomal dominant hereditary spastic paraplegia associated with a novel TUBβ4A mutation. Clin. Genet..

[65] Orlacchio A., Montieri P., Babalini C., Gaudiello F., Bernardi G., Kawarai T. (2011). Late-onset hereditary spastic paraplegia with thin corpus callosum caused by a new SPG3A mutation. J. Neurol..

[66] Lu X., Yang M., Yang Y., Wang X.F. (2020). Atlastin-1 modulates seizure activity and neuronal excitability. CNS Neurosci. Ther..

[67] Salinas S., Proukakis C., Crosby A., Warner T.T. (2008). Hereditary spastic paraplegia: Clinical features and pathogenetic mechanisms. Lancet Neurol..

[68] Andersen E.W., Leventer R.J., Reddihough D.S., Davis M.R., Ryan M.M. (2016). Cerebral palsy is not a diagnosis: A case report of a novel atlastin-1 mutation. J. Paediatr. Child Health.

[69] Rainier S., Sher C., Reish O., Thomas D., Fink J.K. (2006). De novo occurrence of novel SPG3A/atlastin mutation presenting as cerebral palsy. Arch. Neurol..

[70] Yonekawa T., Oya Y., Higuchi Y., Hashiguchi A., Takashima H., Sugai K., Sasaki M. (2014). Extremely severe complicated spastic paraplegia 3A with neonatal onset. Pediatr. Neurol..

[71] Dalpozzo F., Rossetto M.G., Boaretto F., Sartori E., Mostacciuolo M.L., Daga A., Bassi M.T., Martinuzzi A. (2003). Infancy onset hereditary spastic paraplegia associated with a novel atlastin mutation. Neurology.

[72] Kwon M.J., Lee S.T., Kim J.W., Sung D.H., Ki C.S. (2010). Clinical and genetic analysis of a Korean family with hereditary spastic paraplegia type 3. Ann. Clin. Lab. Sci..

[73] Leonardi L., Marcotulli C., Santorelli F.M., Tessa A., Casali C. (2015). De novo mutations in SPG3A: A challenge in differential diagnosis and genetic counselling. Neurol. Sci..

[74] Wolf S.I., Braatz F., Metaxiotis D., Armbrust P., Dreher T., Döderlein L., Mikut R. (2011). Gait analysis may help to distinguish hereditary spastic paraplegia from cerebral palsy. Gait Posture.

[75] Liebschner D., Afonine P.V., Baker M.L., Bunkóczi G., Chen V.B., Croll T.I., Hintze B., Hung L.W., Jain S., McCoy A.J., Moriarty N.W., Oeffner R.D., Poon B.K., Prisant M.G., Read R.J. (2019). Macromolecular structure determination using X-rays, neutrons and electrons: Recent developments in Phenix. Acta Crystallogr. D Struct. Biol..

[76] Emsley P., Lohkamp B., Scott W.G., Cowtan K. (2010). Features and development of Coot. Acta Crystallogr. D Biol. Crystallogr..

[77] Schneider C.A., Rasband W.S., Eliceiri K.W. (2012). NIH image to ImageJ: 25 years of image analysis. Nat. Methods.

[78] Svenstrup K., Bross P., Koefoed P., Hjermind L.E., Eiberg H., Born A.P., Vissing J., Gyllenborg J., Nørremølle A., Hasholt L., Nielsen J.E. (2009). Sequence variants in SPAST, SPG3A and HSPD1 in hereditary spastic paraplegia. J. Neurol. Sci..

[79] Haberlová J., Claeys K.G., Zamecnik J., De Jonghe P., Seeman P. (2008). Extending the clinical spectrum of SPG3A mutations to a very severe and very early complicated phenotype. J. Neurol..

[80] Chen S.Q., Zhou Y., Li X.Y., La B., Huang S., Huang W.J., Zhou C.L., Maxwell P.H., Wang Y.M. (2005). Severe hereditary spastic paraplegia caused by a de novo SPG3A mutation. Sci. China.

[81] Álvarez V., Sánchez-Ferrero E., Beetz C., Díaz M., Alonso B., Corao A.I., Gámez J., Esteban J., Gonzalo J.F., Pascual-Pascual S.I., López de Munain A., Moris G., Ribacoba R., Márquez C., Rosell J. (2010). Mutational spectrum of the SPG4 (SPAST) and SPG3A (ATL1) genes in Spanish patients with hereditary spastic paraplegia. BMC Neurol..

[82] Dürr A., Camuzat A., Colin E., Tallaksen C., Hannequin D., Coutinho P., Fontaine B., Rossi A., Gil R., Rousselle C., Ruberg M., Stevanin G., Brice A. (2004). Atlastin1 mutations are frequent in young-onset autosomal dominant spastic paraplegia. Arch. Neurol..

[83] Lu X., Cen Z., Xie F., Ouyang Z., Zhang B., Zhao G., Luo W. (2014). Genetic analysis of SPG4 and SPG3A genes in a cohort of Chinese patients with hereditary spastic paraplegia. J. Neurol. Sci..

[84] D’Amico A., Tessa A., Sabino A., Bertini E., Santorelli F.M., Servidei S. (2004). Incomplete penetrance in an SPG3A-linked family with a new mutation in the atlastin gene. Neurology.

[85] Elert-Dobkowska E., Stepniak I., Krysa W., Rajkiewicz M., Rakowicz M., Sobanska A., Rudzinska M., Wasielewska A., Pilch J., Kubalska J., Lipczynska-Lojkowska W., Kulczycki J., Kurdziel K., Sikorska A., Beetz C. (2015). Molecular spectrum of the SPAST, ATL1 and REEP1 gene mutations associated with the most common hereditary spastic paraplegias in a group of Polish patients. J. Neurol. Sci..

[86] Ishiura H., Takahashi Y., Hayashi T., Saito K., Furuya H., Watanabe M., Murata M., Suzuki M., Sugiura A., Sawai S., Shibuya K., Ueda N., Ichikawa Y., Kanazawa I., Goto J. (2014). Molecular epidemiology and clinical spectrum of hereditary spastic paraplegia in the Japanese population based on comprehensive mutational analyses. J. Hum. Genet..

[87] Varga R.E., Schüle R., Fadel H., Valenzuela I., Speziani F., Gonzalez M., Rudenskaia G., Nürnberg G., Thiele H., Altmüller J., Alvarez V., Gamez J., Garbern J.Y., Nürnberg P., Zuchner S. (2013). Do not trust the pedigree: Reduced and sex-dependent penetrance at a novel mutation hotspot in ATL1 blurs autosomal dominant inheritance of spastic paraplegia. Hum. Mutat..

[88] Magariello A., Tortorella C., Citrigno L., Patitucci A., Tortelli R., Mazzei R., Conforti F.L., Ungaro C., Sproviero W., Gambardella A., Muglia M. (2012). The p.Arg416Cys mutation in SPG3a gene associated with a pure form of spastic paraplegia. Muscle Nerve.

[89] De Leva M.F., Filla A., Criscuolo C., Tessa A., Pappatà S., Quarantelli M., Bilo L., Peluso S., Antenora A., Longo D., Santorelli F.M., De Michele G. (2010). Complex phenotype in an Italian family with a novel mutation in SPG3A. J. Neurol..

