# Supplementary material for: A novel insertion mutation in atlastin 1 is associated with spastic quadriplegia, increased membrane tethering, and aberrant conformational switching
Source: J Biol Chem. 2021 Nov 19;298(1):101438. doi: 10.1016/j.jbc.2021.101438 (PMC8688574; doi:10.1016/j.jbc.2021.101438)
Supplement: Supplemental Figures S1–S3 [file mmc1.pdf]

Supporting information for manuscript:

A novel insertion mutation in Atlastin 1 is associated with spastic quadriplegia, increased membrane tethering, and aberrant conformational switching

Carolyn M. Kelly[1,#], Peter J. Zeiger[1], C4RCD Research Group[2], Vinodh Narayanan[2], Keri Ramsey[2,#], Holger Sondermann[1,3,4,#]

[1] Department of Molecular Medicine, College of Veterinary Medicine, Cornell University, Ithaca, NY 14853, USA
[2] Center for Rare Childhood Disorders, Translational Genomics Research Institute (TGen), Phoenix, AZ, USA
[3] CSSB Centre for Structural Systems Biology, Deutsches Elektronen-Synchrotron DESY, Hamburg, Germany
[4] Christian-Albrechts-Universität zu Kiel, Kiel, Germany

[#] To whom correspondence should be addressed:
Email: cmk269@cornell.edu
Email: kramsey@tgen.org, phone: +1 602 687 8193
Email: holger.sondermann@cssb-hamburg.de, phone: +49 40 8998 87680

<u>List of materials included:</u>

Figure S1. Colocalization of ATL1 with an ER marker in U2OS cells. (Supplement to Figure 2).
Figure S2. Vesicle tethering controls. (Supplement to Figure 5).
Figure S3. Limited proteolysis controls. (Supplement to Figure 6).

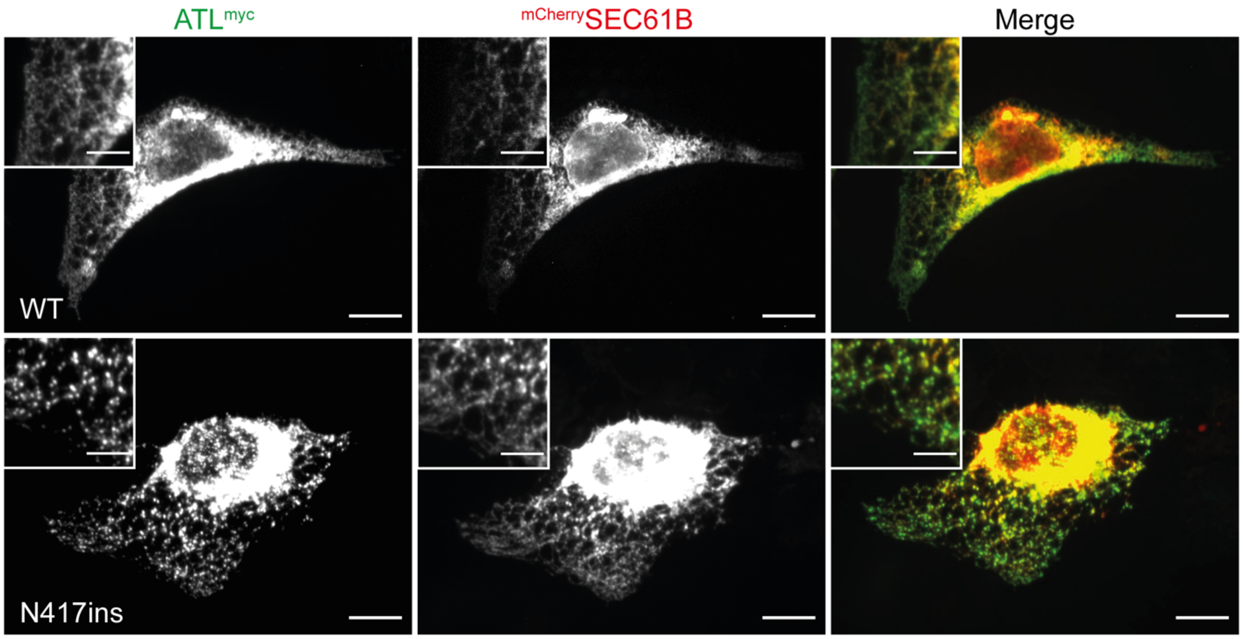

Figure S1. Colocalization of ATL1 with an ER marker in U2OS cells. Immunofluorescence was carried out on U2OS cells transiently co-transfected with ^{mCherry}SEC61B and either ATL1 WT^{myc} (top) or ATL1 N417ins^{myc} (bottom). The first column shows ATL1 signal (green), the second column shows SEC61B (red), and the third column overlays the signals. Scale bars = 10 µm; inset scale bar = 5 µm.

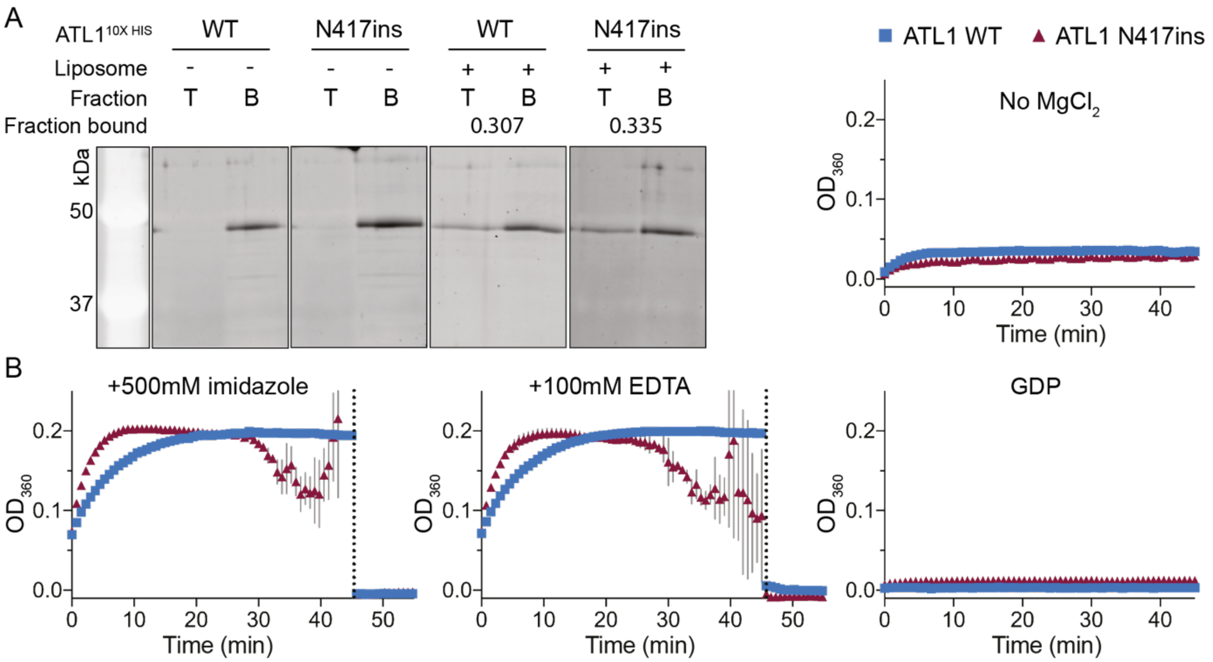

Figure S2. Vesicle tethering controls. (A) Flotation assay with 1 μM ATL1 WT or N417ins catalytic core proteins with a C-terminal His_{10}-tag in the presence (right two panes) or absence (left two panes) or lipids containing 1% molar ratio of Ni-NTA-modified lipids. Top fraction (indicated by "T") contains vesicles and bottom fraction ("B") contains unbound protein. For conditions with lipids, the fraction of protein bound to lipids was calculated using ImageJ (77). (B) Tethering reactions with 1 μM protein including addition of 10 mM EDTA or 500 mM imidazole at 45 minutes, or in the absence of $MgCl_2$ or presence of 2 mM GDP.

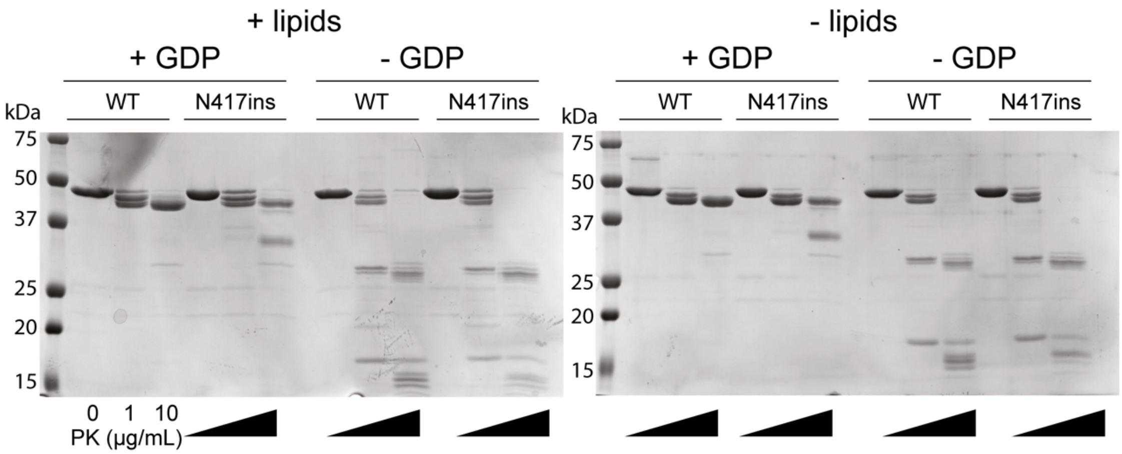

Figure S3. Limited proteolysis controls. SDS-PAGE gels show limited proteolysis reactions as described in Figure 6, but in the presence or absence of liposomes as prepared for vesicle tethering assays (see Figure 5). Reactions were carried out with increasing concentrations of PK (0 to 10 µg/mL) as indicated at the bottom of the gel and by the black triangles.

References cited in supporting information

77. Schneider, C. A., Rasband, W. S., and Eliceiri, K. W. (2012) NIH Image to ImageJ: 25 years of image analysis. *Nat. Methods* **9**, 671-675.
